# Quantitative Singlet Fission in Solution-Processable Dithienohexatrienes

**DOI:** 10.1021/jacs.2c10254

**Published:** 2022-12-28

**Authors:** Kealan J. Fallon, Nipun Sawhney, Daniel T. W. Toolan, Ashish Sharma, Weixuan Zeng, Stephanie Montanaro, Anastasia Leventis, Simon Dowland, Oliver Millington, Daniel Congrave, Andrew Bond, Richard Friend, Akshay Rao, Hugo Bronstein

**Affiliations:** † Department of Chemistry, University of Cambridge, Cambridge CB2 1EW, U.K.; ‡ Cavendish Laboratory, University of Cambridge, Cambridge CB3 0HE, U.K.; § Department of Chemistry, University of Sheffield, Dainton Building, Brook Hill, Sheffield S3 7HF, U.K.

## Abstract

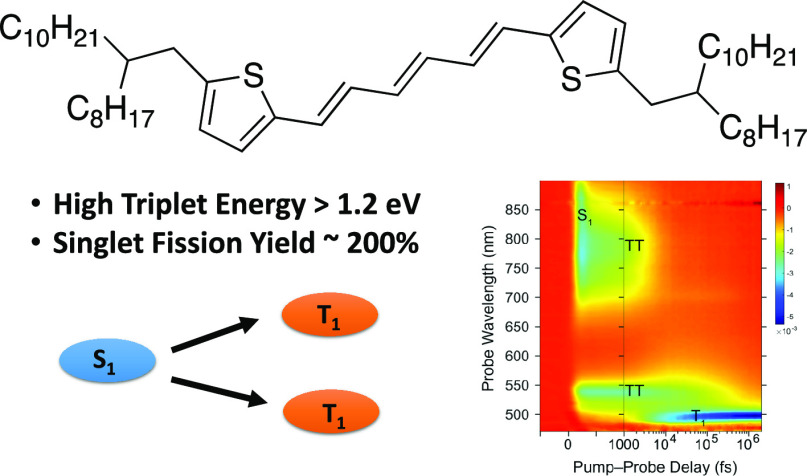

Singlet fission (SF) is a promising strategy to overcome thermalization losses and enhance the efficiency of single junction photovoltaics (PVs). The development of this field has been strongly material-limited, with a paucity of materials able to undergo SF. Rarer still are examples that can produce excitons of sufficient energy to be coupled to silicon PVs (>1.1 eV). Herein, we examine a series of a short-chain polyene, dithienohexatriene (DTH), with tailored material properties and triplet (T_1_) energy levels greater than 1.1 eV. We find that these highly soluble materials can be easily spin-cast to create thin films of high crystallinity that exhibit ultrafast singlet fission with near perfect triplet yields of up to 192%. We believe that these materials are the first solution-processable singlet fission materials with quantitative triplet formation and energy levels appropriate for use in conjunction with silicon PVs.

## Introduction

Photovoltaic (PV) technologies that enable the generation of electricity from sunlight are among the most promising for meeting the large-scale demand expected of global economies as the world transitions toward a more sustainable future. Silicon PV cells are a global commercial success, with modern single-junction efficiencies reaching 26%; however, they are rapidly approaching their thermodynamic limit (ca. 29%).^1^ Emerging technologies in early stages of development that can overcome or effectively raise this limit offer exciting pathways of innovation in this area.^2^ One promising strategy is to address a major facet of the fundamental losses that limit the PV cell: thermalization.^3^ When a PV absorbs a high-energy photon, the portion of energy in excess of the bandgap of the PV (e.g., Si 1.1 eV) is wasted as heat. To overcome this issue, high-energy photons could be more efficiently captured through multiexcitonic processes, where the photon energy could be split into two excitons within an optical material.

One such process in organic molecules is singlet fission (SF).^4^ Here, a high-energy singlet exciton generated from the absorption of a photon by an organic molecule can interact with a neighboring ground-state molecule to form two triplet excitons.^5^ If the resulting triplet excitons produced from singlet fission have energy greater than the bandgap of the PV cell, then they can be harvested as a photocurrent.^6^ Theoretical analysis suggests that the limit of a single-junction silicon PV cell can be raised up to ca. 44% by employing SF.^7^

This realization has triggered a surge of interest in singlet fission over the past decade, and great advancements have been made, especially in the elucidation of the often complex intermediate states that facilitate the phenomenon.^8−13^ Despite these efforts, there remains a paucity of materials able to undergo singlet fission, and moreover, even rarer are examples that can produce triplet states with sufficient energy to be coupled to Si (1.1 eV).^14^ A marginal offset with Si is likely to be beneficial to ensure efficient transfer of excitations (either directly or via emissive methods),^6,15^ meaning that a practical range for the ideal SF material triplet energy level is 1.2–1.4 eV, and considering the requirement E(S_1_) ≥ 2E(T_1_), this limits the S_1_ energy to the range of 2.4–2.8 eV for an idealized material.^15^ Additionally, solution processability is another crucial material requirement to enable facile application of the required large area thin films during manufacture.

Unlike the field of thermally activated delayed fluorescence (TADF), where robust design rules have been elucidated for achieving a small singlet–triplet gap,^16^ there currently exists no simple strategy for generating materials with large S_1_–T_1_ gaps on the order required for SF, and this topic is an exciting and rapidly developing area of investigation and debate.^17−19^ Furthermore, the engineering of large S_1_–T_1_ gaps in organic materials that also exhibit wide optical gaps where S_1_ >2 eV is extremely challenging. Many emerging novel SF systems are large polycyclic aromatic hydrocarbons with substantial π-systems, which narrow the optical gap to below the desired level for coupling to Si.^20^ A few exceptions are present such as 9,10-bis(phenylethynyl)anthracene, which has a T_1_ energy between 1.1 and 1.2 eV in the solid state and undergoes SF in high yield but cannot be solution-processed due to its low solubility. Perylenediimides also have reported T_1_ ∼1.1 eV and can undergo efficient singlet fission but lack sufficient energetic offset to be practically useful in conjunction with Si technology. Finally, tetracene and diphenylhexatriene both possess sufficiently high T_1_ energy (≥1.2 eV) but lack solution processability.^6,21−23^ Thus, there are essentially no solution-processable singlet fission materials with suitable energy levels for use with Si that give triplet quantum yields >100%. This means that a practical application of SF cannot yet be realized. Therefore, there is an urgent requirement for the development of organic materials that can both undergo efficient singlet fission and produce high-energy triplet excited states while maintaining solution processability.

Herein, we explore the potential of short-chain polyenes as idealized SF candidates. Inspired by reports of efficient singlet fission in carotenoid-type aggregates and single crystals of oligoenes,^17,23,24^ we design a family of novel dithienohexatriene (DTH) materials through efficient and scalable synthesis. The materials exhibit excellent solubility and processability. Using ultrafast transient absorption spectroscopy, we find that ultrafast singlet fission is active in all spin-cast films with the choice of alkyl chain clearly impacting the kinetics of triplet migration and recombination. The photophysical characteristics are correlated to the crystallinity in the films as observed by X-ray scattering, showcasing the breadth of control of SF yield and triplet lifetime through molecular engineering. Thus, we believe that we report the first solution-processable singlet fission material that may be of practical use in conjunction with Si PV technologies.

## Results and Discussion

**Figure 1:**
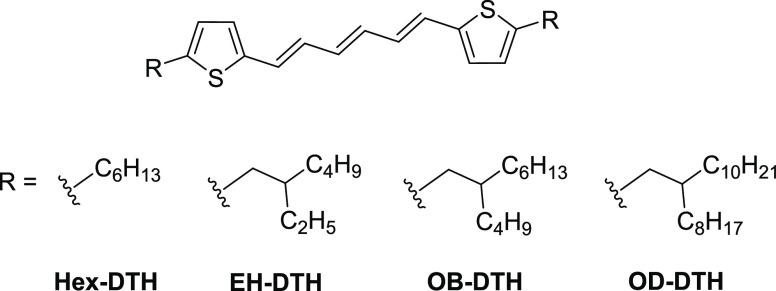
Structures of the four alkylated dithienohexatrienes in this study: hexyl-DTH (**Hex–DTH**); 2-ethylhexyl (**EH–DTH**); 2-octylbutyl (**OB–DTH**); 2-octyldodecyl (**OD–DTH**).

Dithienohexatriene (DTH, Figure 1 where R = H) can be synthesized through a one-pot Horner–Wadsworth–Emmons reaction of 2 equivalents of thenaldehyde and tetraethyl-but-2-ene-1,4-diyl(*E*)-bis(phosphonate) in ca. 50% yield.^25^

**Figure 2:**
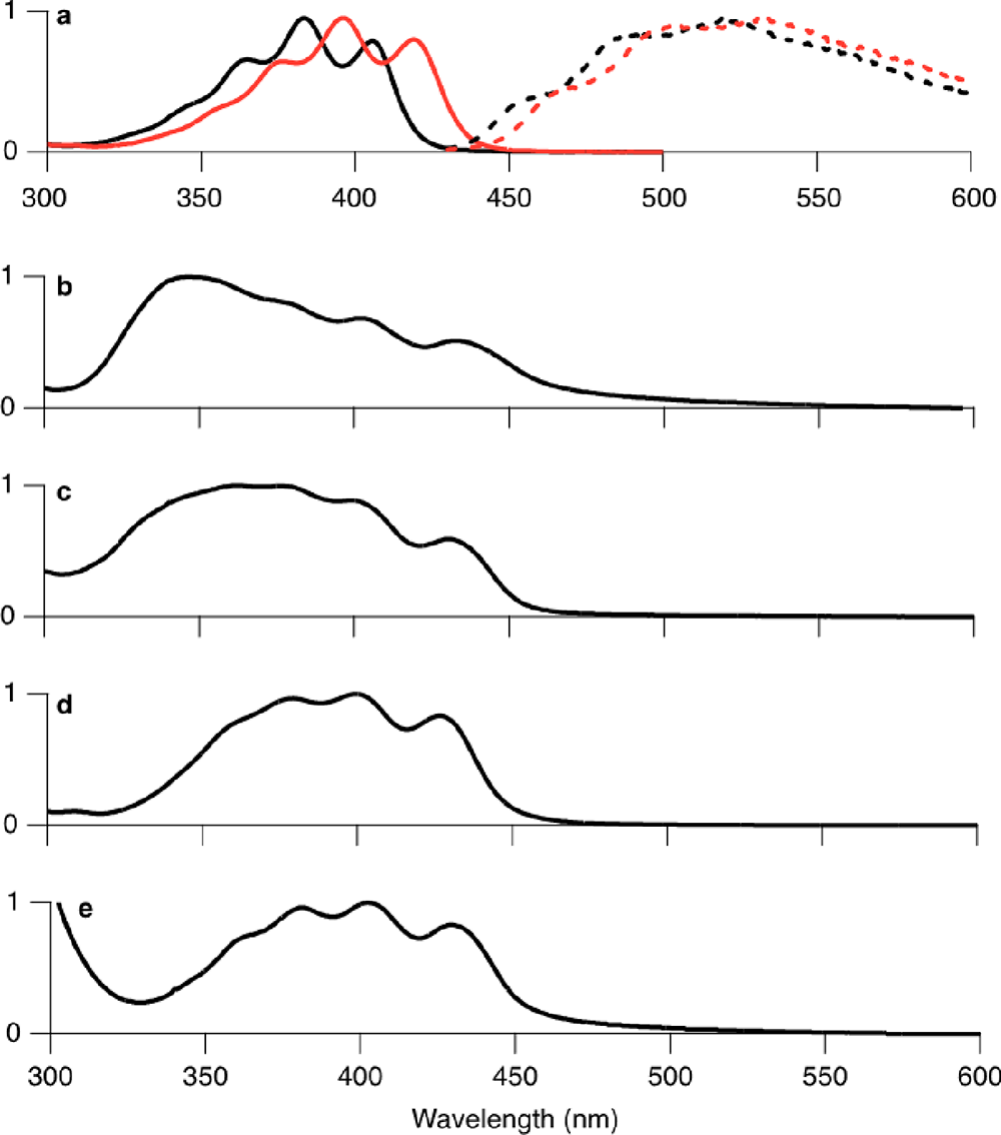
(a) Normalized absorption (solid lines) and emission (dashed lines) spectra of dilute solutions of DTH (black) and **EH–DTH** (red); normalized absorption spectra of thin films (spin-coated from CHCl_3_ at 15 mg/mL) of (b) **Hex–DTH**, (c) **EH–DTH**, (d) **OB–DTH**, and (e) **OD–DTH**.

Unsubstituted dithienohexatriene is a highly insoluble yellow powder. We sought to improve the solubility and processability of the chromophore through the installation of solubilizing alkyl side chains. We therefore prepared a family of 2-thiophenecarboxaldehydes with a variety of alkyl chains installed at the 5-position (see the Supporting Information, Section S1). DTH functionalized with linear hexyl chains (**Hex–DTH**) showed improved solubility but remained highly crystalline, undergoing spontaneous crystallization in saturated solutions and upon drop-casting. Longer, branched alkyl chains as expected resulted in materials with excellent solubility even at high concentrations. All four alkylated materials were highly processible through typical spin-casting techniques from typical organic solvents. In dilute solution (chloroform), all four materials exhibited identical absorption and emission characteristics (Figure 2a), slightly bathochromically shifted relative to unsubstituted DTH. The solution absorption spectra of the alkylated materials show a clear vibronic progression, with pronounced 0–0, 0–1, and 0–2 transitions and a fourth 0–3 as a slight shoulder feature. In thin film, all four materials exhibited a broadening of their absorption, characteristic of the interaction of organic molecules in the solid state, but shared similar absorption onsets in all four films, slightly red-shifted (∼20 nm) relative to the solution measurements (Figure 2). The vibronic structure is also visible for all four films; however, there is a contribution in the blue region of the spectrum (ca. 340 nm) indicative of H-type aggregation. This contribution is significant for **Hex–DTH**, dominating the absorption profile. As the alkyl chain length increases from EH< OB < OD, the intensity of this absorption is weakened, suggesting suppression of this aggregate; indeed, in **OD–DTH**, the aggregate is not observed. Despite the difference in spectral fine structure, all materials display absorption onsets at ∼450 nm (∼2.75 eV), meaning that their singlet energies are in the ideal range for an SF absorber. We can, with reasonable accuracy, estimate the T_1_ energy of the new materials by comparison of experimental vs calculated values to be within the range of 1.2–1.4 eV (Supporting Information, Section S2), ratifying their potential as ideal SF candidates.

Finally, we note the extensive literature and complicated nature of oligoene lowest excited states regarding the exact positioning of the ^2^A_g_ and ^1^B_u_ states. Both the absorption and photoluminescence spectra have similar appearances to the wider bandgap diphenylexatriene, so we can tentatively assign the excited state ordering to be similar, with S_2_ being the optically bright ^1^Bu state with a lower lying, multiconfigurational ^2^A_g_ state.^26^

**Figure 3:**
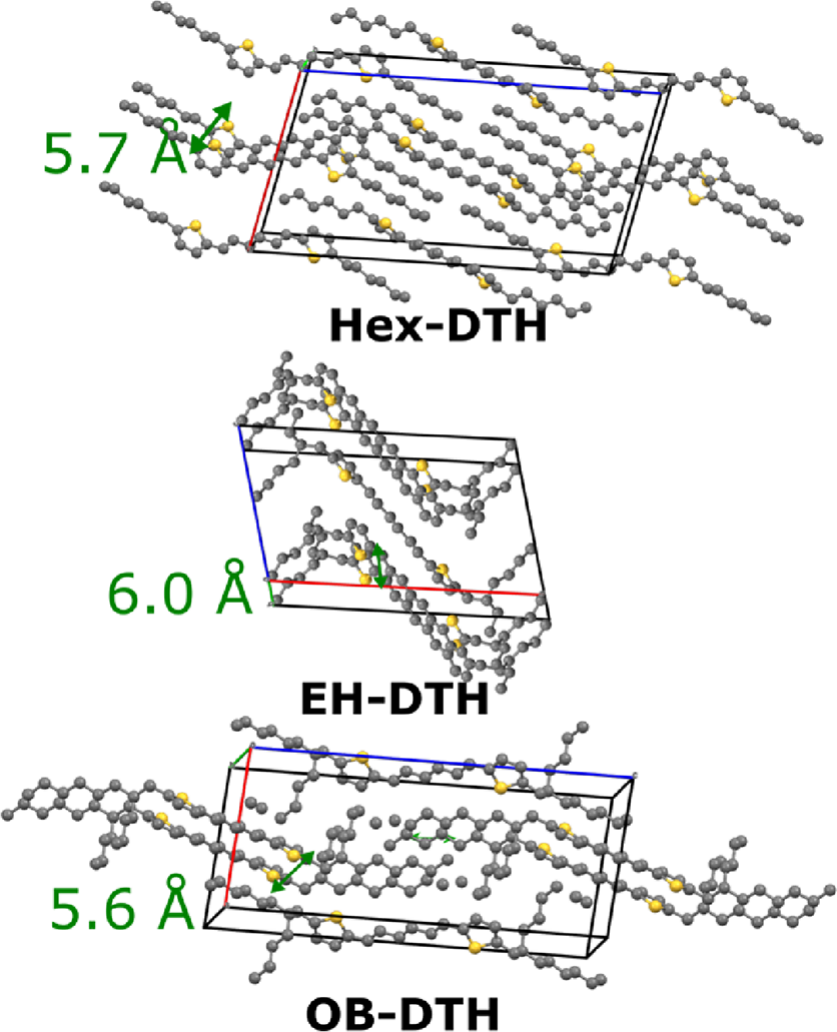
Crystal structures of **Hex–DTH** (CCDC number: 2179619), **EH–DTH** (CCDC number: 2179618), and **OB–DTH** (CCDC number: 2179620), including measured distances between the DTH backbones.

Single crystals (Figure 3 and Supporting Information, Section S3) were successfully grown through solvent evaporation techniques for the three shorter chain molecules (it was not possible to obtain crystals of **OD–DTH** due to its high solubility in most solvents). Two out of the three crystal structures (Supporting Information, Section S3), namely, **Hex–DTH** and **OB–DTH**, show face-to-face slip-stacked columns with approximately identical relative positions of the DTH cores, with a π–π distance of 5.6–5.7 Å. Both **Hex–DTH** and **OB–DTH** adopt brickwork-type packing motifs, in which interactions between the π–π stacked columns are screened by alkyl chains from the molecules in adjacent π–π stacks. In **Hex–DTH**, this leads to column separations of ca. 8 Å, while in **OB–DTH**, the increased size of the alkyl solubilizing groups leads to larger column separations of ca. 11 Å. By contrast, the crystal structure of **EH–DTH** shows a lamella-type structure, in which layers composed of the DTH cores alternate with layers composed of the ethylhexyl groups. Within the DTH layers, the molecules adopt a herring-bone type pattern, forming edge-to-face arrangements with a dihedral angle of ca. 50° between the conjugated DTH cores. Such a pattern is similar to that seen in the crystal structure of unsubstituted DTH, although the molecules in **EH–DTH** are shifted laterally relative to each other and spaced more widely to accommodate the packing requirements of the alkyl chains. Clearly, the 2D side-on nature of the DTH core packing in **EH–DTH** is significantly different from the 1D columns seen in **Hex–DTH** and **OB–DTH**.

**Figure 4:**
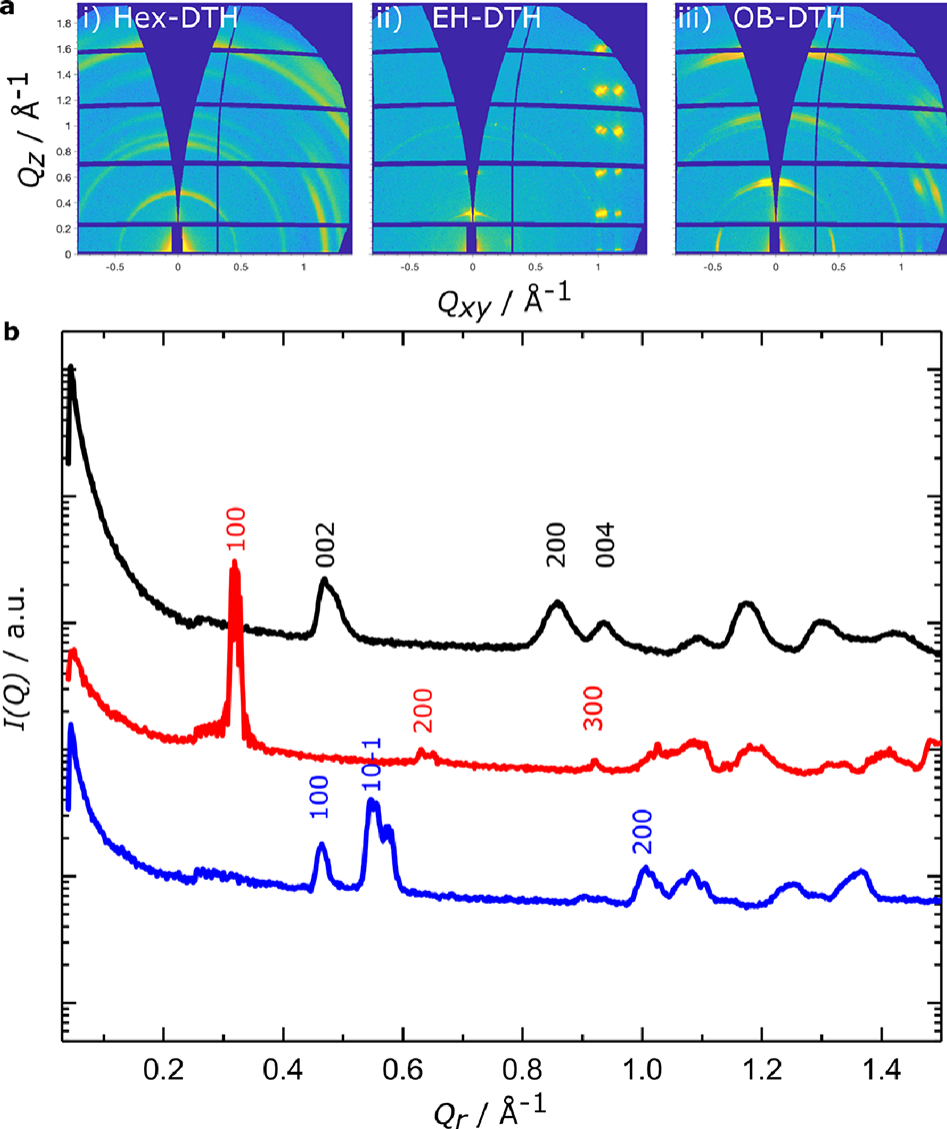
(a) Two-dimensional grazing incidence X-ray scattering of (i) **Hex–DTH**, (ii) **EH–DTH**, and (iii) **OB–DTH** thin films and corresponding 1D radially integrated data (b) for **Hex–DTH** (black), **EH–DTH** (red), and **OB–DTH** (blue), with data scaled for clarity and scattering peaks indexed according to single-crystal structures.

To investigate the microstructure of the three thin films in greater detail, grazing incidence wide-angle X-ray scattering (GIWAXS) was performed on spin-cast films prepared on silicon substrates, with two-dimensional and corresponding radial integrated scattering data presented in Figure 4. For the **Hex–DTH** film, the 2D scattering patterns comprise Debye–Scherrer rings, indicating that the crystalline morphology consists of many large domains of randomly oriented crystallites. **OB–DTH** is largely similar to **Hex–DTH**; however, not all angles are expressed equally, with some preferential ordering of crystallites. The **EH–DTH** film exhibits clear preferential ordering of the X00 lamella scattering peaks in the *Q_z_* axis and so are orientated in-plane. Additionally, the array of strong Bragg spots at *Q_xy_* ∼1 to 1.2 Å^–1^ and spaced at regular intervals along the *Q_z_* indicates that the thin-film crystal structure is highly ordered, with relatively large crystal grains. For all the films studied herein, indexing of the radially integrated scattering data (*Q*_r_) presented in Figure 4b indicates that the thin films adopt the same crystalline arrangement as the aforementioned single-crystal structures.

**Figure 5:**
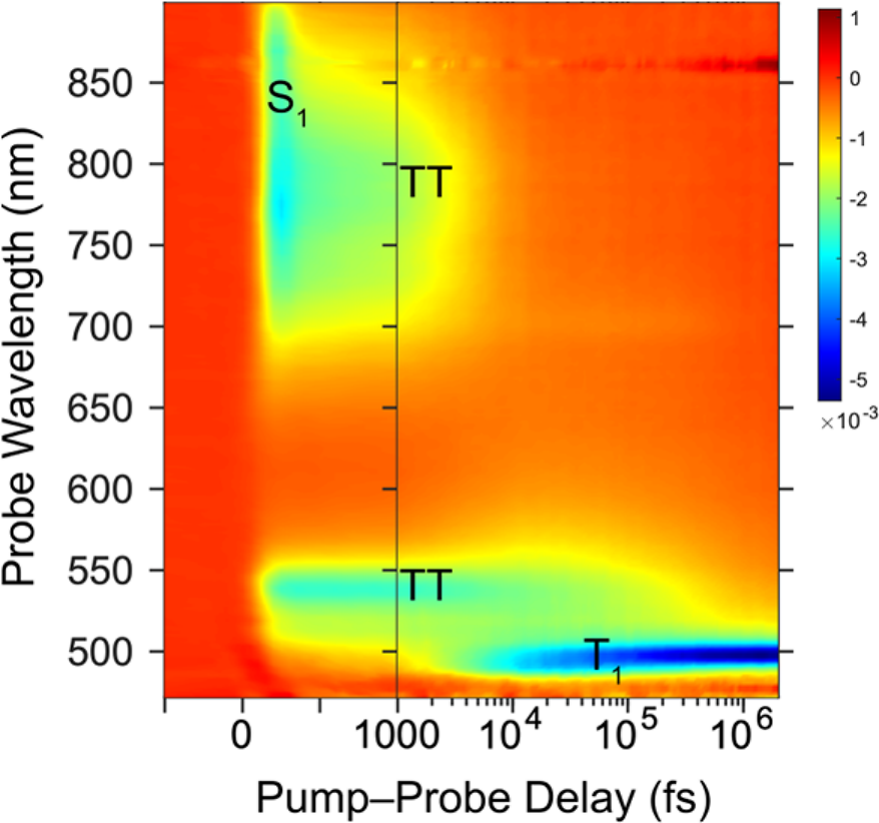
Transient absorption of **Hex–DTH** in the fs–ns regime when pumped at 430 nm. The negative signal (Δ*T*/*T*) corresponds to the absorption of photoexcited states (labeled).

To determine the characteristics of the excited state dynamics of these spin-cast films, we used transient absorption (TA) spectroscopy. Here, the sample is excited with a short narrowband laser pulse (pump) and then interrogated with a broadband pulse (probe) at a controllable time delay (50 fs to 1 ms). Changes in the transmission of the probe (Δ*T*/*T*) constitute the absorption spectrum of photoexcited states, also known as photoinduced absorption (PIA), depopulation of the ground state, also known as ground state bleach (GSB), and stimulated emission (SE). Full details of the experiment and equipment are available in the Supporting Information, Section S5. The TA spectra of **Hex–DTH** in the fs–ps regime are shown in Figure 5. The corresponding spectra for **EH–DTH** and **OB–DTH** are shown in Figure S13.

In the first picosecond, PIA of the initially formed singlet excited state is observed around 550 nm and broadly in the near infrared (NIR) between 700 and 900 nm. Between 1 and 10 ps, there is a red-shift in the PIA suggesting a transition to a slightly different state—one we will loosely assign to the TT state and will discuss in more depth below. After 10 ps, an intense sharp PIA of a new excited state emerges between 480 and 500 nm, becoming the dominant feature in all three films within 100 ps. This species is assigned as the triplet (T_1_) state as its characteristics correlate strongly with the sensitized triplet signature, as shown in Section S5 of the Supporting Information.

**Figure 6:**
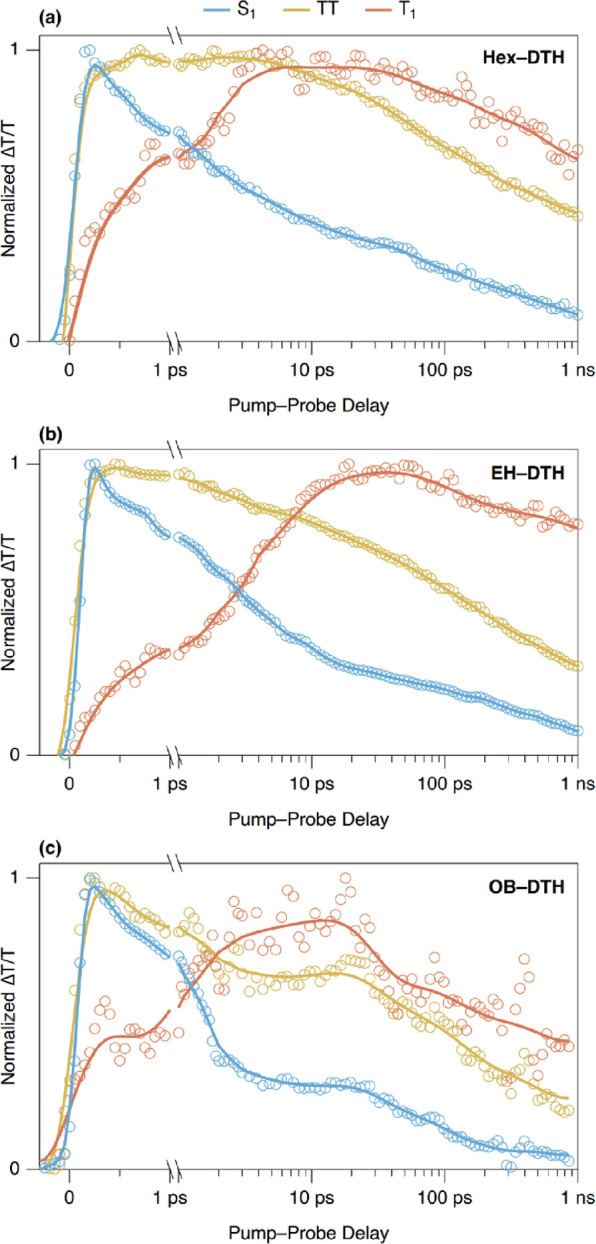
Kinetics of the three excitonic species observed in the fs–ns TA spectra when pumped at 430 nm: the singlet state (S_1_, blue, 700–750 nm), the coupled triplet pair state (TT, yellow, 520–540 nm), and free triplet states (T_1_, orange, 480–500 nm) for (a) **Hex–DTH**, (b) **EH–DTH**, and (c) **OB–DTH**.

Furthermore, this state exhibits a long lifetime into the ns regime, well beyond that of a singlet state, and is not observed on unencapsulated thin films exposed to oxygen. The kinetics of the formation and decay of these three excited state species across all three material films is shown in Figure 6.

The singlet state rises within the instrument response time (100 fs) in all three films and decays monoexponentially in **Hex–DTH** and biexponentially in **EH–DTH** and **OB–DTH**. It is widely accepted that the conversion between the singlet and triplet states in singlet fission proceeds through a coupled triplet pair with the overall singlet character, i.e., ^1^(TT).^11^ There is evidence of a TT state in these films, which has very similar PIA to the initially formed singlet state but slightly red-shifted in absorption characteristics. We observe that the TT rise is complete by 1 ps in all films. The rise of the free triplet state (T_1_) correlates well to the decay of the TT state in all films. Triplet formation is significantly faster in **Hex–DTH** versus the other two films, which exhibit similar kinetics.

**Figure 7:**
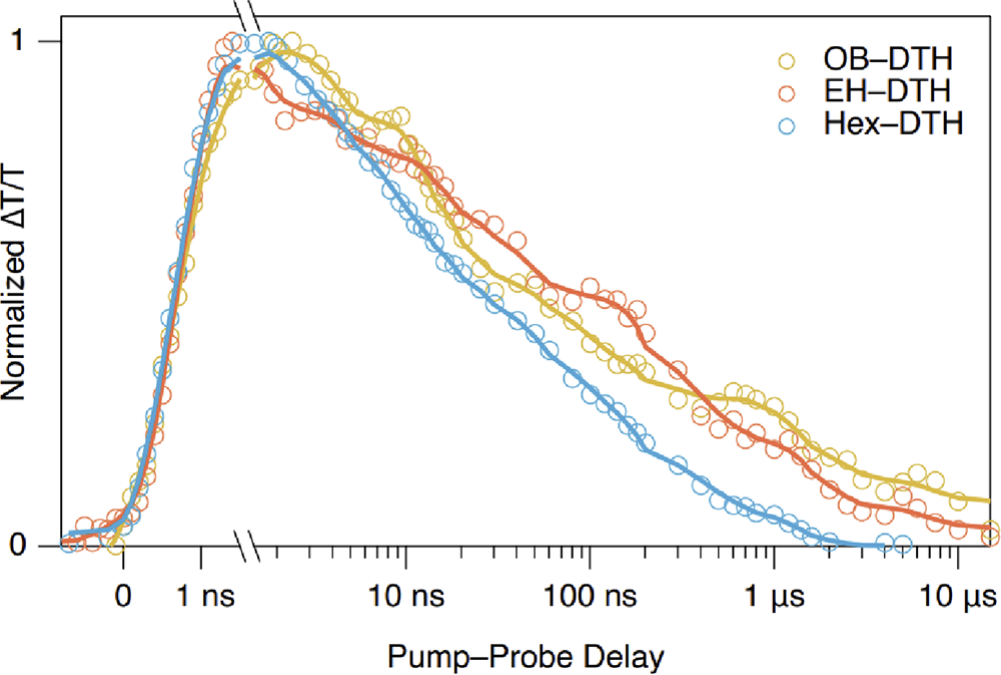
Kinetics of the decay of the PIA corresponding to the free triplet states (T_1_) in the three DTH films.

To understand the decay of the triplet state in better detail, the TA spectra of the films in the ns−μs time domain were examined (Figure 7). Furthermore, the lifetimes of the excited state species were calculated using singular value decomposition of the spectra (see the Supporting Information, Section S5) and are shown in Table 1.

**Table 1:** Characteristics of Singlet Fission in DTH Films

	excited state lifetimes			
material	S_1_	TT	T_1_	singlet fission yield
**Hex–DTH**	1140 fs	90 ps	160 ns	117%
**EH–DTH**	2150 fs	172 ps	370 ns	78%
**OB–DTH**	2800 fs	177 ps	220 ns	193%

The T_1_ state decays fastest in **Hex–DTH** films (160 ns) and is longest lived in **EH–DTH** (370 ns). Singlet fission yields are calculated using triplet cross sections gathered from sensitization experiments (as shown in the Supporting Information, Section S5). From this analysis, we estimate the quantum yield of singlet fission QY(SF) to be highest in **OB–DTH** (192%) and lower in the other two films, 117 and 78% for **Hex** and **EH**, respectively. The SF efficiency of **OB–DTH**, the most efficient of these materials, is similar to those of tetracene and other tetracene derivatives (e.g., TIPS-Tetracene) and is essentially quantitative.^9^ In **OD–DTH** films, due to the material’s low melting point, the thin-film material in the focus of the lasers tended to melt during analysis (Figure S15), and while it appears that this material also undergoes singlet fission, we were unable to extract conclusive or quantitative data from this material.

The correlation of the ultrafast photophysics with the X-ray and single-crystal analysis offers interesting insights. The rates and yields of singlet fission in materials are often found to be controlled by the extent of electronic coupling between two chromophores in the crystallites. In the crystal structures, the DTH cores are relatively far away from each other, and as such, the electronic coupling between adjacent chromophores is likely to be small. This is consistent with the minimal red-shift between the solution and thin-film absorption spectra of all the materials. This is in contrast to unsubstituted DTH that is much closer packed and as a result undergoes significant red-shifting upon crystallization.^25^ The preference for **EH–DTH** to arrange in a lamellar packing motif with side-on contacts between the DTH cores contrasts with **Hex–DTH** and **OB–DTH**, in which the molecules adopt slip-stacked columns. It appears that such lamellar packing in films of DTH can lead to longer triplet lifetimes. Additionally, lamellar packing is compatible (at least in this case) with large, ordered crystal domains in the bulk film. These combined features result in a favorable environment for the triplets, able to migrate through the highly ordered 2D bulk. In the case of **Hex–DTH** and **OB–DTH**, it is notable that the 1D DTH columns within the crystal are geometrically almost identical, so the interaction between chromophores would be expected to be closely comparable, but the fission yield differs greatly. A very small difference is seen in the slip distance between chromophores, and this has been suggested to greatly affect rates and yields of singlet fission in other materials. Careful analysis of the crystal structure also reveals a slight undulation in the oligoene backbone for **OB–DTH**. Preliminary calculations on dimers extracted from the single crystal suggest that these subtle differences may be sufficient to result in H-like aggregate behavior in **Hex–DTH** but not in **OB–DTH**, consistent with the thin-film absorption measurements and may explain the difference in singlet fission yields. These results highlight the importance of controlling and understanding the solid-state packing of organic materials, where changes in crystal packing can have dramatic consequences for the photophysics.

## Conclusions

The coupling of singlet fission materials to silicon photovoltaics can only be realized by the development of a processable singlet fission material that (i) provides T_1_ excitons with energy in the range of 1.2–1.4 eV, (ii) undergoes high efficiency SF, and (iii) can be applied in high-throughput, scalable, thin-film technologies. Herein, we report a series of tailored dithienohexatrienes containing specific alkyl chains that exhibit high solubility and can be spin-cast to produce thin films of high crystallinity. We find that singlet fission is active in all DTH films with yields up to 192%, and the kinetics of the excited state dynamics correlate well to the crystallinity observed within the thin films. Importantly, the singlet and triplet energies of these short-chain aromatic molecules are in the idealized range (S_1_ 2.4–2.8 eV and T_1_ 1.2–1.4) and thus are arguably the most promising candidates ever reported for straightforward coupling to commercial Si PV technologies. Furthermore, these molecules can be synthesized in high quantities through relatively straightforward methods, creating a fertile new ground for exploring the development of a new generation of industrially relevant singlet fission materials.

## Supporting Information Available

Full synthetic procedures along with additional spectroscopy and calculations (PDF)
