# Supplementary material for: Quantitative Singlet Fission in Solution-Processable Dithienohexatrienes
Source: J Am Chem Soc. 2022 Dec 28;144(51):23516–21. doi: 10.1021/jacs.2c10254 (PMC9801381; doi:10.1021/jacs.2c10254)
Supplement: Supplementary file 1 — ja2c10254_si_001.pdf [file ja2c10254_si_001.pdf]

Quantitative Singlet Fission in Solution Processable Dithienohexatrienes

Kealan J. Fallon, ‡[a,b] Nipun Sawhney, ‡[b] Daniel T. W. Toolan,[c] Ashish Sharma,[b] Weixuan, Zeng,[a] Stephanie Montanaro,[a] Anastasia Leventis,[a] Simon Dowland,[b] Oliver Millington,[a] Daniel Congrave,[a] Andrew Bond,[a] Richard Friend,[b] Akshay Rao,*[b] Hugo Bronstein*[a,b]

[a]Department of Chemistry, University of Cambridge, Cambridge, CB2 1EW, U.K.

[b]Cavendish Laboratory, University of Cambridge, Cambridge, CB3 0HE, U.K.

[c]Department of Chemistry, University of Sheffield, Dainton Building, Brook Hill, Sheffield, S3 7HF, U.K.

S1 Materials....2

Methods....2

Synthesis & characterisation....2

S2 Estimation of Triplet Energy....12

S3 Single Crystal X-ray Diffraction....14

S4 Grazing Incidence Small Angle X-ray Scattering....20

S5 Transient Absorption Spectroscopy....21

Sensitization of EH-DTH....21

Calculation of Singlet Fission Yield....22

Error Calculation....24

Solution Concentration Series of DEH-DTH....25

Pump Probe Spectroscopy of DTH Films....26

S6 Analysis of electronic interactions in single crystal structure....29

TD-DFT Analysis of transitions within dimers in single crystal geometry....32

S7 Stability....33

S8 References....35

S1 Materials

Methods

Unless otherwise stated, reactions were performed under an atmosphere of argon using commercial precursors used as received without purification. Reactions in anhydrous solvents were performed in oven-dried glassware, cooled under vacuum on a Schlenk line and backfilled with argon. Anhydrous solvent was purchased directly from suppliers and used without additional drying procedures.

^{1}H NMR spectra were recorded at 400 MHz on a Bruker 400 Neo Prodigy Spectrometer in the stated solvent using residual protic solvent chloroform-d (δ = 7.26, s) or methanol-d_4 (δ = 3.31, s) as the internal standard. Chemical shifts (δ) are quoted in ppm using the following abbreviations: s, singlet; d, doublet; t, triplet; q, quartet; qn, quintet; m, multiplet; br, broad; or a combination of these. Coupling constants (*J*) are reported in hertz (Hz).

^{13}C NMR spectra were recorded at 100 MHz on a Bruker 400 Neo Prodigy Spectrometer in the stated solvent using the central reference of chloroform-d (δ = 77.0, t) or methanol-d_4 (δ = 49.0, s) as the internal standard. Chemical shifts are reported to the nearest 0.1 ppm.

High resolution mass spectroscopy were obtained using a Waters LCT Premier Spectrometer.

Synthesis & characterisation

Friedel–Crafts acylation of thiophene

To a solution of thiophene (1 equiv.) in anhydrous DCM (1 M) cooled with an ice bath was added an acyl chloride (1 equiv.) followed by portionwise addition of $AlCl_3$ (1.5 equiv.). The reaction was allowed to warm naturally to RT and stir overnight. The mixture was then quenched by carefully pouring into cold water. The organic layer was separated from the aqueous and reduced under vacuum to an oil which was dissolved in adequate diethyl ether and extracted with water (2×), brine (2×), dried over $MgSO_4$ and concentrated *in vacuo* to give a crude oil that was used in subsequent reactions without purification.

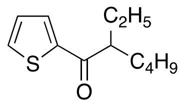

2-Ethyl-1-(thiophen-2-yl)hexanone *in 79% yield*

^{1}H NMR (400 MHz, $CDCl_3$) δ 7.72 (dd, *J* = 3.8, 1.1 Hz, 1H), 7.63 (dd, *J* = 4.9, 1.1 Hz, 1H), 7.13 (dd, *J* = 4.9, 3.8 Hz, 1H), 3.13 (tt, *J* = 8.2, 5.4 Hz, 1H), 1.85 – 1.71 (m, x2H), 1.63 – 1.47 (m, 2H), 1.34 – 1.20 (m, 4H), 0.87 (dt, *J* = 14.3, 7.2 Hz, 6H) **^{13}C NMR** (100 MHz, $CDCl_3$) δ 197.5, 145.6, 133.6, 131.5, 128.1, 50.1, 32.2, 29.8, 25.9, 22.9, 13.9, 12.1 **HRMS** Found (ASAP+): $[M+H]^+$ 211.1160, $C_{12}H_{19}SO$ requires 211.1157

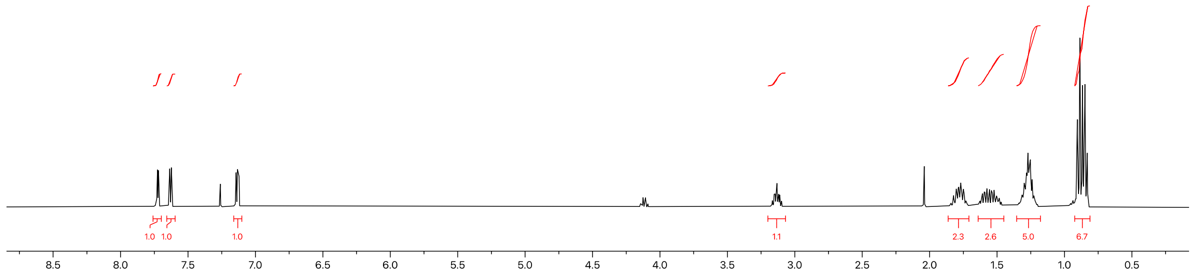

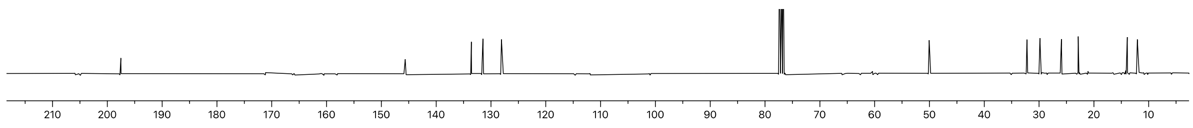

2-Butyl-1-(thiophen-2-yl)octanone *in 88% yield*

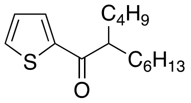

^{1}H NMR (400 MHz, $CDCl_3$) δ 7.72 (dd, *J* = 3.8, 1.1 Hz, 1H), 7.63 (dd, *J* = 4.9, 1.1 Hz, 1H), 7.13 (dd, *J* = 5.0, 3.8 Hz, 1H), 3.19 (tt, *J* = 8.3, 5.4 Hz, 1H), 1.83 – 1.68 (m, 2H), 1.57 – 1.42 (m, 2H), 1.37 – 1.14 (m, 12H), 0.85 (t, *J* = 7.0 Hz, 6H) **^{13}C NMR** (100 MHz, $CDCl_3$) δ 197.7, 145.6, 133.6, 131.5, 128.1, 48.6, 33.0, 32.7, 31.6, 29.9, 29.4, 27.7, 22.9, 22.6, 14.0, 13.9 **HRMS** Found (ASAP+): $[M+H]^+$ 267.1790, $C_{16}H_{27}SO$ requires 267.1783

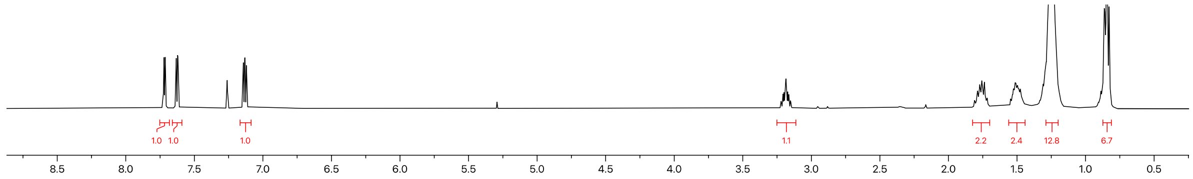

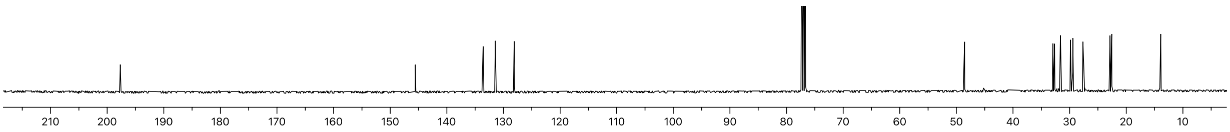

1-(Thiophen-2-yl)dodecanone *in 72% yield*

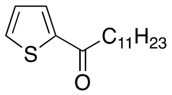

^{1}H NMR (400 MHz, $CDCl_3$) δ 7.70 (dd, *J* = 3.8, 1.1 Hz, 1H), 7.61 (dd, *J* = 5.0, 1.1 Hz, 1H), 7.12 (dd, *J* = 4.9, 3.8 Hz, 1H), 2.88 (t, *J* = 7.5 Hz, 2H), 1.74 (p, *J* = 7.4 Hz, 2H), 1.43 – 1.20 (m, 16H), 0.87 (t, *J* = 6.8 Hz, 3H) **^{13}C NMR** (100 MHz, $CDCl_3$) δ 193.6, 144.5, 133.3, 131.6, 128.0, 39.5, 31.9, 29.6, 29.5, 29.4, 29.3, 24.8, 22.7, 14.1 **HRMS** Found (ASAP+): $[M+H]^+$ 267.1793, $C_{16}H_{27}SO$ requires 267.1783

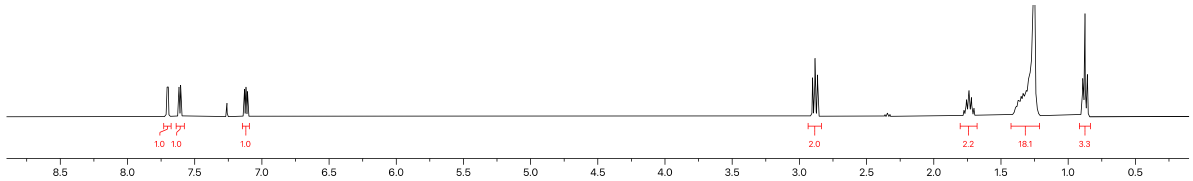

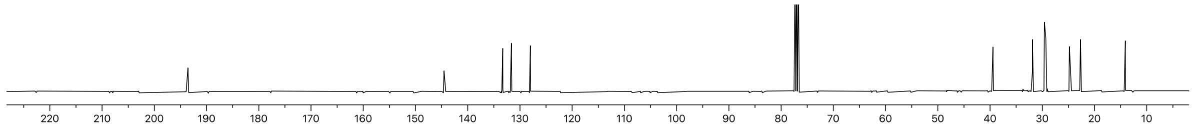

~ ~ ~

Wolff–Kishner reduction of thienyl ketones

Under rigorous exclusion of oxygen, a solution of a thienyl ketone (1 equiv.) in diethylene glycol (0.25 M) and hydrazine hydrate (65%, 5 equiv.) were heated to 180 °C in distillation apparatus, collecting by distillation excess hydrazine and water. After 2 h, the setup was cooled to 80 °C, the still head replaced with a condenser, potassium hydroxide (4 equiv.) pellets

added at once, and the reaction heated to 195 °C. After 3 h the reaction was cooled, poured into water and extracted with diethyl ether (3×). The combined organic extracts were washed with water (2×), brine (2×), dried over $MgSO_4$ as concentrated *in vacuo*. The crude oil was purified by flash chromatography eluting with hexane.

2-(2-Ethylhexyl)thiophene *in 53% yield, colourless oil*

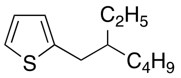

^{1}H NMR (400 MHz, $CDCl_3$) δ 7.12 (dd, *J* = 5.1, 1.2 Hz, 1H), 6.93 (dd, *J* = 5.1, 3.4 Hz, 1H), 6.78 (dd, *J* = 3.4, 1.1 Hz, 1H), 2.78 (d, *J* = 6.9 Hz, 2H), 1.65 – 1.55 (m, 1H), 1.43 – 1.25 (m, 8H), 0.91 (t, *J* = 7.4 Hz, 6H) **^{13}C NMR** (100 MHz, $CDCl_3$) δ 144.4, 126.5, 125.0, 122.9, 41.5, 33.8, 32.4, 28.9, 25.5, 23.0, 14.1, 10.8 **HRMS** Found (ASAP+): $[M+H]^+$ 197.1365, $C_{12}H_{21}S$ requires 197.1364

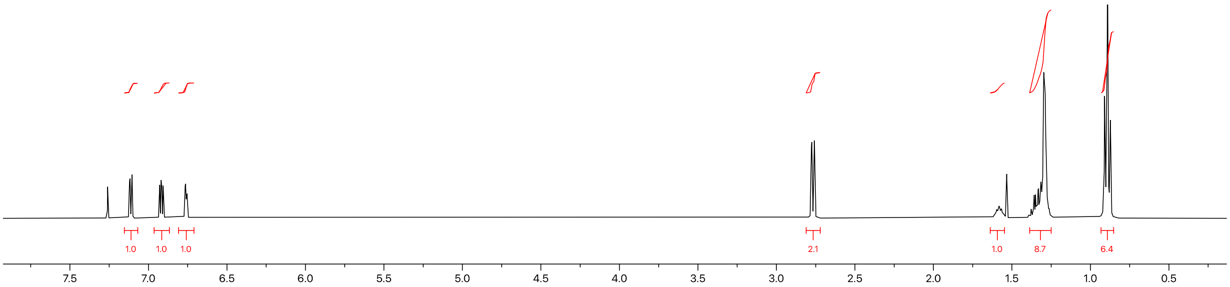

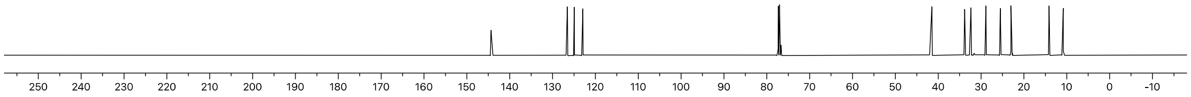

2-(2-Butyloctyl)thiophene *in 59% yield, colourless oil*

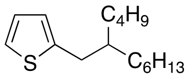

^{1}H NMR (400 MHz, $CDCl_3$) δ 7.12 (dd, *J* = 5.1, 1.2 Hz, 1H), 6.92 (dd, *J* = 5.1, 3.4 Hz, 1H), 6.76 (dd, *J* = 3.4, 1.1 Hz, 1H), 2.77 (d, *J* = 6.7 Hz, 1H), 1.64 (q, *J* = 5.9 Hz, 1H), 1.38 – 1.22 (m, 16H), 0.90 (t, *J* = 6.8 Hz, 6H) **^{13}C NMR** (100 MHz, $CDCl_3$) δ 144.3, 126.5, 125.0, 122.9, 40.0, 34.2, 33.2, 32.9, 31.9, 29.7, 28.8, 26.6, 23.0, 22.7, 14.1 **HRMS** Found (ASAP+): $[M+H]^+$ 253.1990, $C_{16}H_{29}S$ requires 253.1990

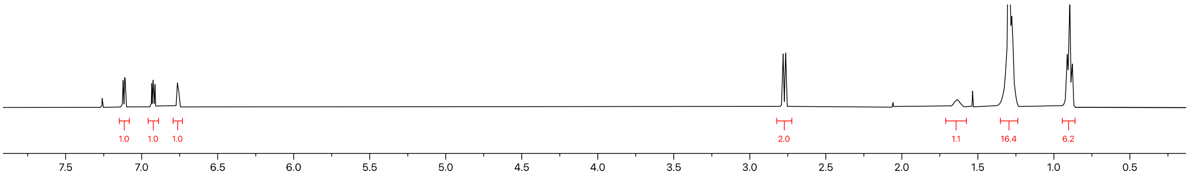

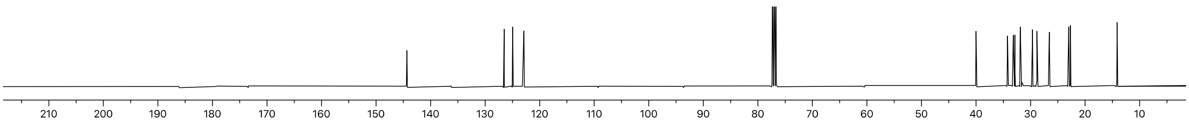

2-Dodecylthiophene *in 64% yield, colourless oil*

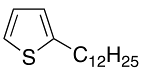

¹H NMR (400 MHz, $CDCl_3$) δ 7.10 (dd, *J* = 5.1, 1.2 Hz, 1H), 6.92 (dd, *J* = 5.1, 3.4 Hz, 1H), 6.79 – 6.76 (m, 1H), 2.82 (t, *J* = 7.7 Hz, 2H), 1.74 – 1.62 (m, 2H), 1.41 – 1.23 (m, 16H), 0.89 (t, *J* = 6.8 Hz, 3H) **¹³C NMR** (100 MHz, $CDCl_3$) δ 145.9, 126.6, 123.9, 122.7, 31.9, 31.8, 31.6, 29.9, 29.7, 29.6, 29.4, 29.1, 22.7, 14.1 **HRMS** Found (ASAP+): $[M+H]^+$ 253.2000, $C_{16}H_{29}S$ requires 253.1990

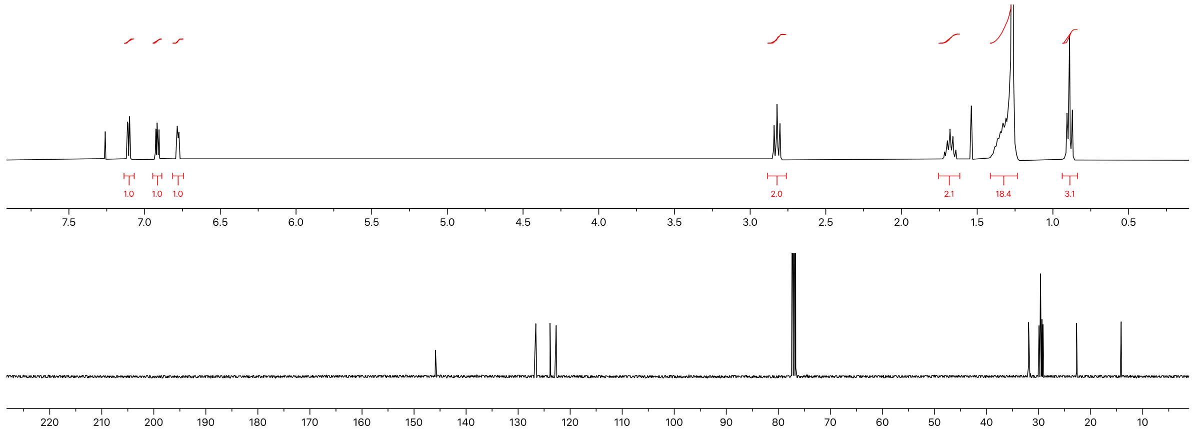

2-(2-Octyldodecyl)thiophene *in 49% yield, colourless oil*

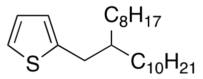

¹H NMR (400 MHz, $CDCl_3$) δ 7.11 (dd, *J* = 5.1, 1.2 Hz, 1H), 6.91 (dd, *J* = 5.1, 3.4 Hz, 1H), 6.77 – 6.73 (m, 1H), 2.76 (d, *J* = 6.6 Hz, 2H), 1.62 (p, *J* = 5.9 Hz, 1H), 1.34 – 1.20 (m, 32H), 0.89 (t, *J* = 6.7 Hz, 6H) **¹³C NMR** (100 MHz, $CDCl_3$) δ 144.4, 126.5, 124.9, 122.9, 40.0, 34.2, 33.2, 31.9, 30.0, 29.7, 29.6, 29.4, 29.3, 26.6, 22.7, 14.1 **HRMS** Found (ASAP+): $[M+H]^+$ 365.3242, $C_{24}H_{45}S$ requires 365.4232

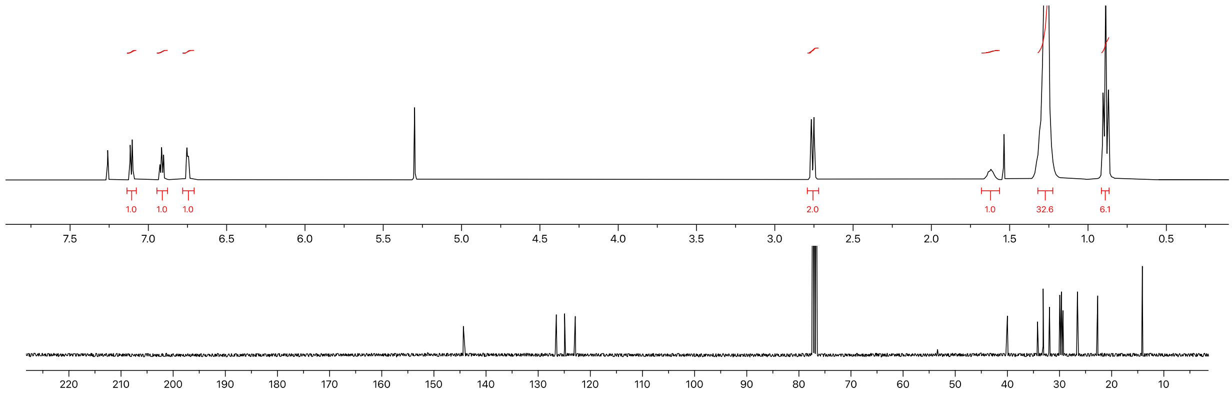

~ ~ ~

Formylation of alkyl thiophenes

To a solution of an alkyl thiophene (1 equiv.) in anhydrous DMF (1.5 M) cooled with an ice bath was added trichlorophosphate (5 equiv.) over 15 minutes. The mixture was then heated to 80 °C for 2 h. After cooling, the mixture was poured carefully into water and stirred rapidly for 1 h. The mixture was extracted with diethyl ether (3×). The combined organic extracts were washed with water (2×), sat. $NaHCO_3$ soln. (2×), dried over

$MgSO_4$ as concentrated *in vacuo*. The crude oil was purified by column chromatography eluting with 0→10% ethyl acetate in hexane.

5-(2-Ethylhexyl)thiophene-2-carbaldehyde *in 53% yield, yellow oil*

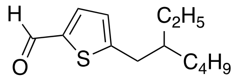

^{1}H NMR (400 MHz, $CDCl_3$) δ 9.81 (s, 2H), 7.61 (d, *J* = 3.8 Hz, 2H), 6.88 (d, *J* = 3.8 Hz, 1H), 2.81 (d, *J* = 6.9 Hz, 2H), 1.68 – 1.56 (m, 1H), 1.39 – 1.22 (m, 8H), 0.91 – 0.85 (m, 6H) **^{13}C NMR** (100 MHz, CDC13) δ 182.6, 156.6, 141.8, 136.9, 126.8, 41.5, 34.8, 32.3, 28.8, 25.5, 22.9, 14.1, 10.8 **HRMS** Found (ASAP+): $[M+H]^+$ 225.1313, $C_{13}H_{21}SO$ requires 225.1313

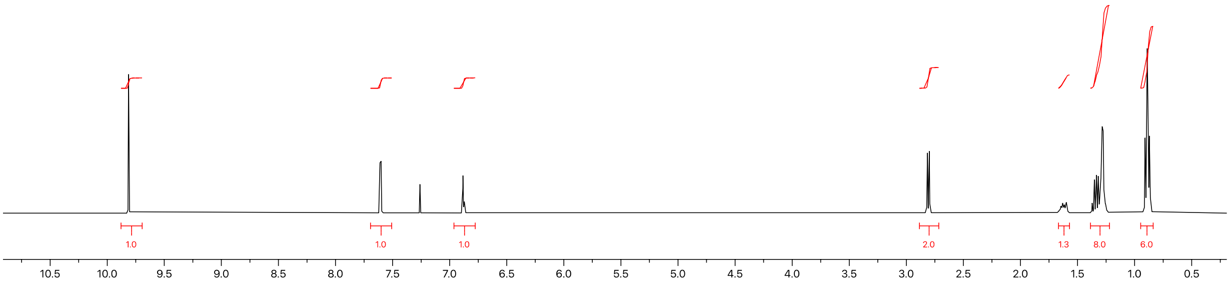

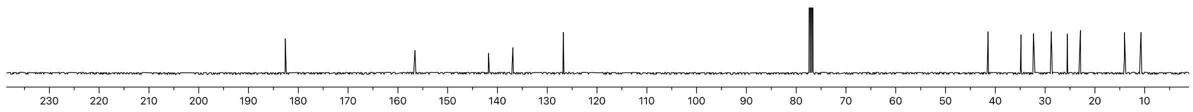

5-(2-Butyloctyl)thiophene-2-carbaldehyde *in 74% yield, yellow oil*

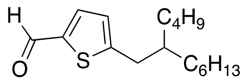

^{1}H NMR (400 MHz, $CDCl_3$) δ 9.81 (s, 1H), 7.61 (d, *J* = 3.7 Hz, 1H), 6.87 (d, *J* = 3.7 Hz, 1H), 2.80 (d, *J* = 6.7 Hz, 2H), 1.72 – 1.62 (m, 1H), 1.34 – 1.19 (m, 17H), 0.91 – 0.83 (m, 6H) **^{13}C NMR** (100 MHz, $CDCl_3$) δ 182.7, 156.6, 141.8, 136.9, 126.8, 40.0, 35.2, 33.2, 32.9, 31.8, 29.5, 28.8, 26.5, 22.9, 22.6, 14.1 **HRMS** Found (ASAP+): $[M+H]^+$ 281.1953, $C_{17}H_{29}SO$ requires 281.1939

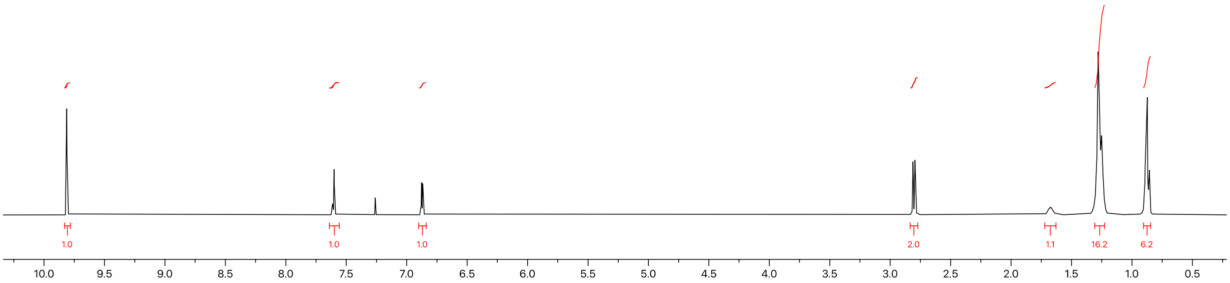

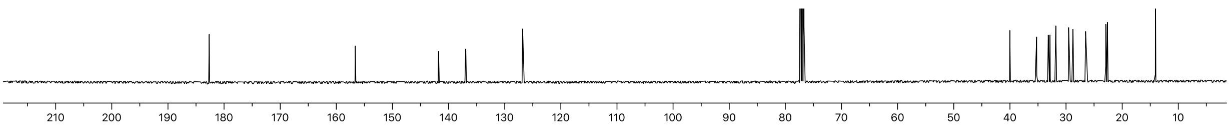

5-Dodecylthiophene-2-carbaldehyde *in 90% yield, yellow oil*

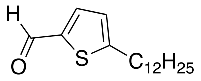

^{1}H NMR (400 MHz, $CDCl_3$) δ 9.81 (s, 1H), 7.60 (d, *J* = 3.8 Hz, 1H), 6.90 (d, *J* = 3.8 Hz, 1H), 2.86 (t, *J* = 7.6 Hz, 2H), 1.76 – 1.65 (m, 2H), 1.41 –

1.21 (m, 18H), 0.92 – 0.84 (m, 3H) **^{13}C NMR** (100 MHz, $CDCl_3$) δ 182.6, 157.8, 141.6, 137.0, 125.8, 31.9, 31.3, 30.8, 29.6, 29.5, 29.3, 29.0, 22.7, 14.1 **HRMS** Found (ASAP+): $[M+H]^+$ 281.1941, $C_{17}H_{29}SO$ requires 281.1939

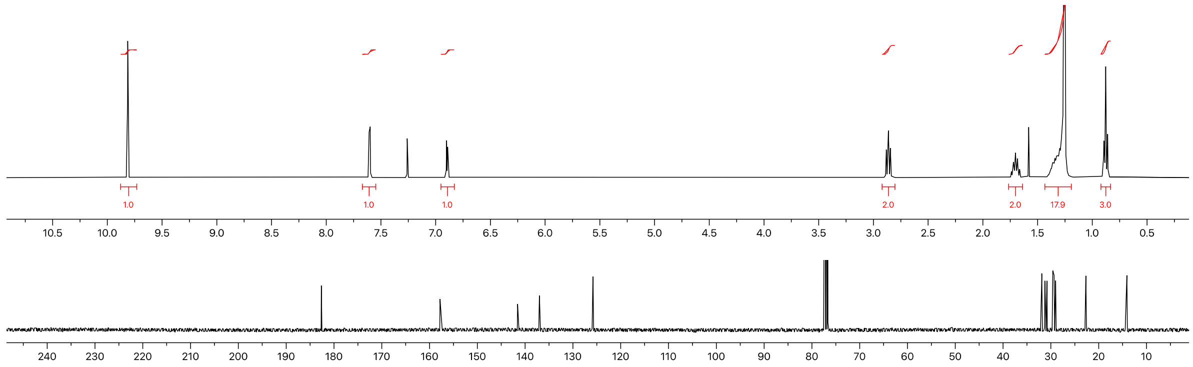

5-(2-Octyldodecyl)thiophene-2-carbaldehyde *in 58% yield, yellow oil*

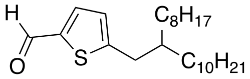

^{1}H NMR (400 MHz, $CDCl_3$) δ 9.82 (s, 1H), 7.61 (d, *J* = 3.8 Hz, 1H), 6.87 (d, *J* = 3.8 Hz, 1H), 2.80 (d, *J* = 6.7 Hz, 1H), 1.74 – 1.62 (m, 1H), 1.34 – 1.18 (m, 32H), 0.90 – 0.85 (m, 6H) **^{13}C NMR** (100 MHz, $CDCl_3$) δ 182.6, 156.6, 141.8, 136.9, 126.8, 40.0, 35.3, 33.2, 31.9, 29.9, 29.6, 29.6, 29.3, 26.5, 22.7, 14.1 **HRMS** Found (ASAP+): $[M+H]^+$ 393.3201, $C_{25}H_{45}SO$ requires 393.3191

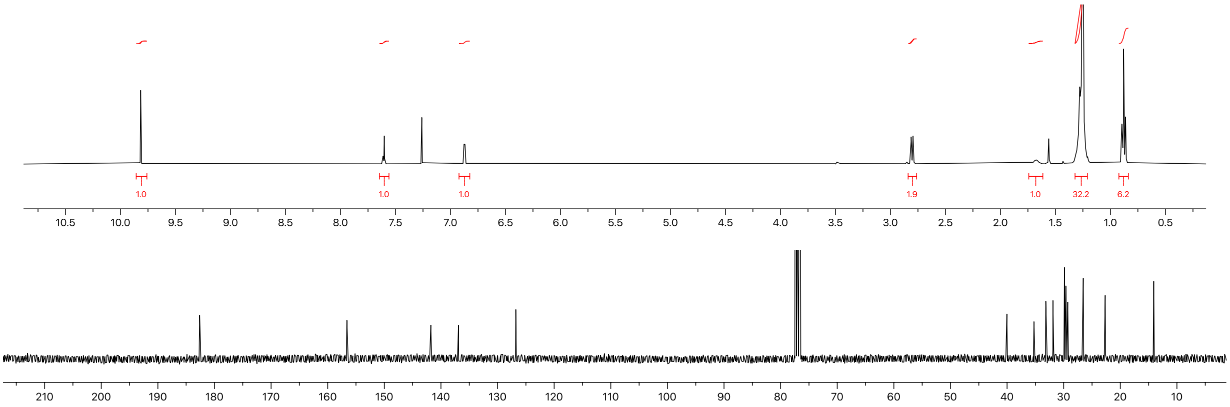

Tetraethyl but-2-ene-1,4-diyl(*E*)-bis(phosphonate)

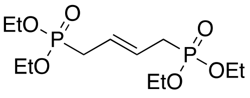

A neat solution of (*E*)-1,4-dibromobutene (25 g, 0.12 mol) in triethyl phosphate (50 mL, 0.29 mol) was heated at 200 °C overnight. The reaction was cooled, and ethyl bromide and some excess phosphate removed *in vacuo*. The neat oil was passed through an inch of celite to remove insoluble impurities and the resulting oil was heated at 150 °C at 8 torr for 30 min, distilling off further impurities. The cooled oil was thus used in subsequent reactions (32 g, pale yellow oil, 83%).

~ ~ ~

Synthesis of dialkyl dithienylhexatrienes

To a rapidly stirred solution of a thiophene carbaldehyde (2.2 equiv.) and phosphonate ## (1 equiv.) in anhydrous THF (0.2 M *to the aldehyde*) was added a freshly prepared solution of potassium *tert*-butoxide (3 equiv.) in anhydrous THF (0.3 M) dropwise in the dark. The reaction was stirred at RT overnight. Water (equal vol. to THF) was added, and the mixture stirred for 1 h.

(1*E*,3*E*,5*E*)-1,6-bis(5-hexylthiophen-2-yl)hexa-1,3,5-triene
viz. **D–DTH**

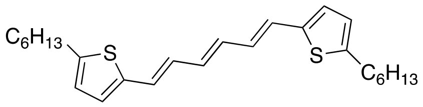

The mixture was extracted with DCM (5×). The combined organic extracts were washed with water (2×), brine (2×), dried over $MgSO_4$ as concentrated *in vacuo*. The crude oil was purified by column chromatography eluting with 10→100% chloroform in hexane, eluting at 70% to give the title compound as a yellow solid in 29% yield.

^{1}H NMR (400 MHz, $CDCl_3$) δ 6.76 (d, *J* = 3.5 Hz, 2H), 6.65 – 6.51 (m, 6H), 6.36 (dd, *J* = 6.6, 3.0 Hz, 2H), 2.76 (t, *J* = 7.6 Hz, 4H), 1.71 – 1.60 (m, 4H), 1.41 – 1.26 (m, 12H), 0.95 – 0.85 (m, 6H) **^{13}C NMR** (100 MHz, $CDCl_3$) δ 140.7, 132.4, 127.9, 125.9, 125.6, 124.7, 31.6, 31.5, 30.4, 28.8, 22.6, 14.1 **HRMS** Found (ASAP+): $[M+H]^+$ 413.2321, $C_{26}H_{37}S_2$ requires 413.2337

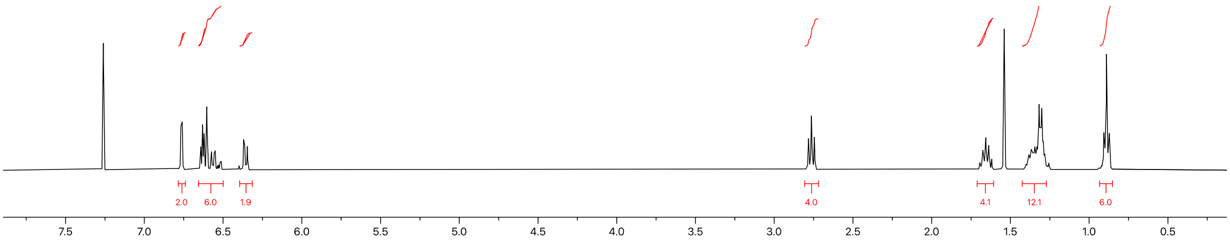

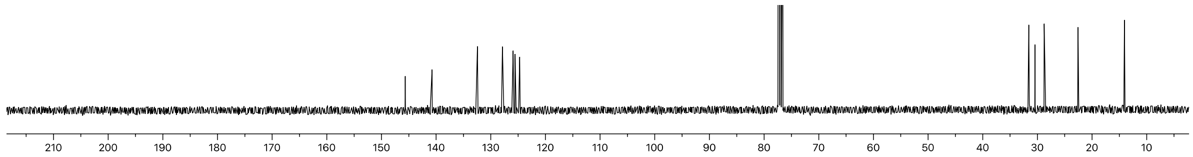

(1*E*,3*E*,5*E*)-1,6-bis(5-hexylthiophen-2-yl)hexa-1,3,5-triene
viz. **EH–DTH**

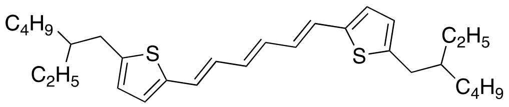

The mixture was extracted with DCM (5×). The combined organic extracts were washed with water (2×), brine (2×), dried over $MgSO_4$ as concentrated *in vacuo*. The crude oil was purified by column chromatography eluting with 10→100% chloroform in hexane, eluting at 70% to give the title compound as a yellow solid in 29% yield.

^{1}H NMR (400 MHz, $CDCl_3$) δ 6.77 (d, *J* = 3.5 Hz, 2H), 6.68 – 6.50 (m, 6H), 6.36 (dd, *J* = 6.5, 3.0 Hz, 2H), 2.71 (d, *J* = 6.7 Hz, 4H), 1.62 – 1.52 (m, 2H), 1.40 – 1.24 (m, 16H), 0.95 – 0.84 (m, 12H) **^{13}C NMR** (100 MHz, $CDCl_3$) δ 144.3, 140.9, 132.4, 127.9, 125.9, 125.8, 125.5, 41.4, 34.4, 32.4, 28.9, 25.5, 23.0, 14.1, 10.8 **HRMS** Found (ASAP+): $[M+H]^+$ 469.2977, $C_{30}H_{45}S_2$ requires 469.2963

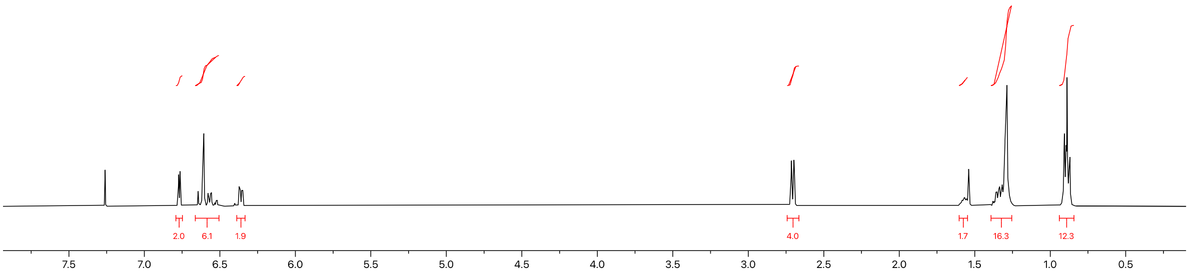

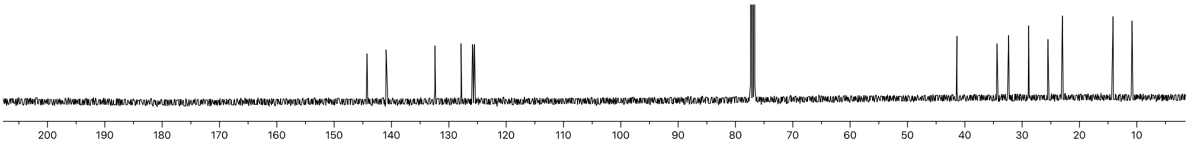

(1*E*,3*E*,5*E*)-1,6-bis(5-(2-butyloctyl)thiophen-2-yl)hexa-1,3,5-triene
viz. **OB–DTH**

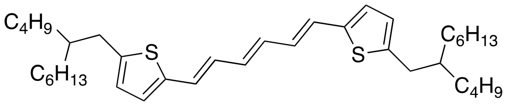

The mixture was extracted with DCM (5×). The combined organic extracts were washed with water (2×), brine (2×), dried over $MgSO_4$ as concentrated *in vacuo*. The crude oil was purified by column chromatography eluting with 10→100% chloroform in hexane, eluting at 30% to give the title compound as a yellow solid in 50% yield.

^{1}H NMR (400 MHz, $CDCl_3$) δ 6.77 (d, *J* = 3.6 Hz, 2H), 6.67 – 6.51 (m, 6H), 6.36 (dd, *J* = 6.5, 3.0 Hz, 2H), 2.70 (d, *J* = 6.6 Hz, 4H), 1.66 – 1.57 (m, 2H), 1.36 – 1.20 (m, 32H), 0.94 – 0.85 (m, 12H) **^{13}C NMR** (100 MHz, $CDCl_3$) δ 144.3, 140.9, 132.4, 127.8, 125.9, 125.8, 125.5, 39.9, 34.8, 33.2, 32.9, 31.9, 29.6, 28.8, 26.6, 23.0, 22.7, 14.1 **HRMS** Found (ASAP+): $[M+H]^+$ 581.4204, $C_{38}H_{61}S_2$ requires 581.4215

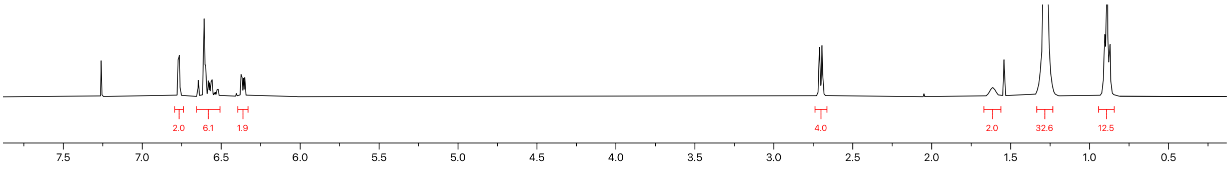

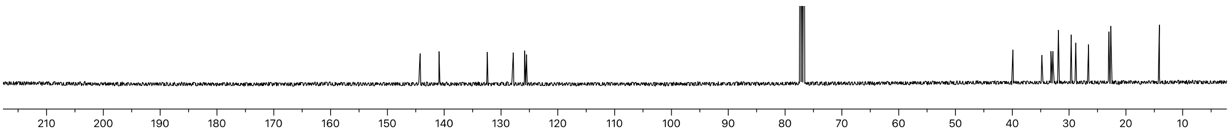

(1*E*,3*E*,5*E*)-1,6-bis(5-dodecylthiophen-2-yl)hexa-1,3,5-triene
viz. **D–DTH**

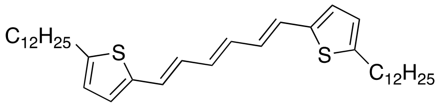

After addition of water, the title compound precipitated from the mixture, which was collected by filtration and washed with minimum diethyl ether then DCM to give a yellow solid which was further dried under high vacuum to afford a yellow solid in 42% yield.

^{1}H NMR (400 MHz, $CDCl_3$) δ 6.76 (d, *J* = 3.5 Hz, 2H), 6.65 – 6.50 (m, 6H), 6.36 (dd, *J* = 6.6, 3.0 Hz, 2H), 2.76 (t, *J* = 7.6 Hz, 4H), 1.65 (p, *J* = 7.5 Hz, 4H), 1.40 – 1.22 (m, 36H), 0.88 (t, *J* = 6.8 Hz, 6H) **^{13}C NMR** (100 MHz, $CDCl_3$) δ 145.7, 140.7, 132.4, 127.9, 125.9, 125.5, 124.7, 31.9, 31.5, 30.4, 29.7, 29.6, 29.5, 29.4, 29.0, 22.7, 14.1 **HRMS** Found (ASAP+): $[M+H]^+$ 580.4144, $C_{38}H_{60}S_2$ requires 580.4136

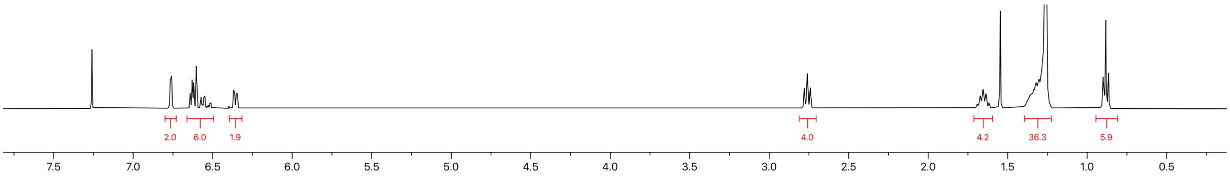

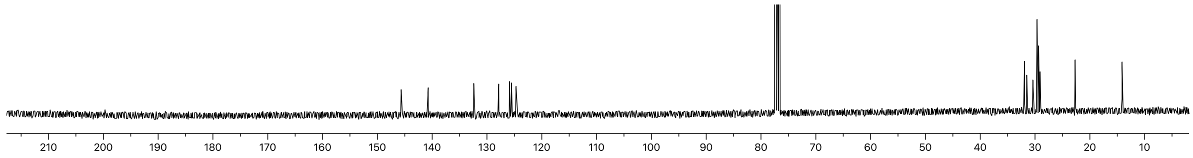

(1*E*,3*E*,5*E*)-1,6-bis(5-(2-butyloctyl)thiophen-2-yl)hexa-1,3,5-triene
viz. **OD–DTH**

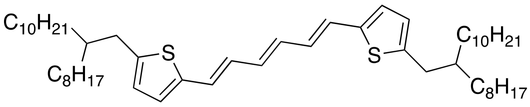

The mixture was extracted with DCM (3×) and the combined organic extracts were washed with water (2×), brine (2×), dried over $MgSO_4$ and concentrated *in vacuo*. The crude oil was purified by column chromatography eluting with hexane to afford a residue, which was recrystallised from acetone with a few drops of ethyl acetate to bring the hot solution to homogeneity followed by cooling in a freezer overnight to give the title compound as a bright yellow waxy solid.

^{1}H NMR (700 MHz, $CDCl_3$) δ 6.77 (d, *J* = 3.5 Hz, 2H), 6.64 – 6.52 (m, 6H), 6.38 – 6.34 (m, 2H), 2.70 (d, *J* = 6.6 Hz, 4H), 1.61 (q, *J* = 6.0 Hz, 2H), 1.34 – 1.20 (m, 64H), 0.88 (t, *J* = 7.0 Hz, 12H) **^{13}C NMR** (175 MHz, $CDCl_3$) δ 144.3, 141.0, 132.4, 127.9, 125.9, 125.8, 125.5, 39.9, 34.8, 33.2, 32.0, 31.9, 30.0, 29.7, 29.6, 29.4, 29.3, 26.6, 22.7, 14.2 **HRMS** Found (ASAP+): $[M+H]^+$ 805.6726, $C_{54}H_{93}S_2$ requires 805.6719

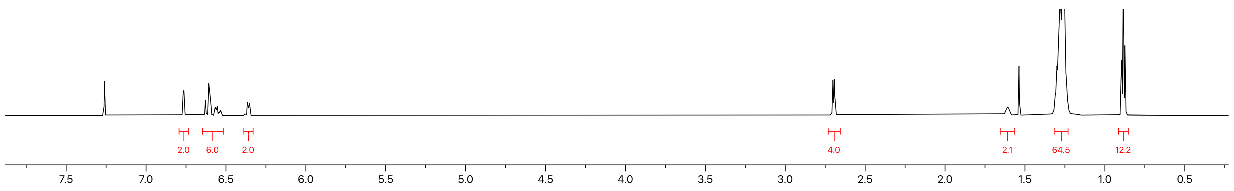

2.0
6.0
2.0
4.0
2.1
64.5
12.2
7.5 7.0 6.5 6.0 5.5 5.0 4.5 4.0 3.5 3.0 2.5 2.0 1.5 1.0 0.5

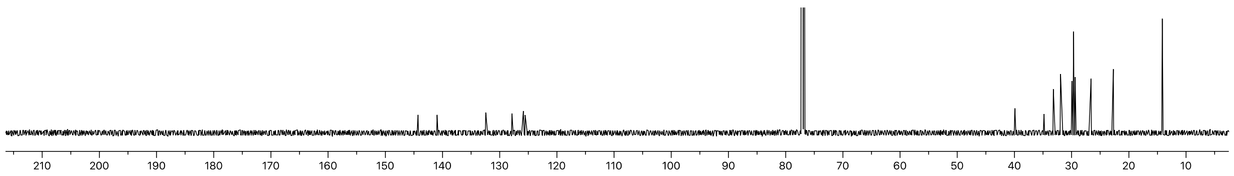

210 200 190 180 170 160 150 140 130 120 110 100 90 80 70 60 50 40 30 20 10

S2 Estimation of Triplet Energy

Structure optimization and excited state analysis

Ground state geometries are optimized at B3LYP/def2-SVP level. The dispersion correction was conducted by Grimme's D3 version by the Gaussian 16 program. Basing on the optimized ground state geometries, the vertical excitation energies were evaluated at M06-2X/def2-TZVP by TD-DFT method with polarizable continuum model (PCM) taking the dielectric constant for toluene ($\varepsilon = 2.37$) and chloroform ($\varepsilon = 4.71$) as reference. Excited states analysis was processed with the TDDFT results using Multiwfn 3.8 program according to the program manual and literature method. The $E(T_1)$'s were also evaluated with a ΔSCF procedure at DFT M06-2X/def2-SVP level, which manually adjusts the spin multiplicities for both vertical and adiabatic geometries. Key values were collected as **Figure S1** and **Table S1**.

The computation results show that the DTH and its alkyl substituted derivatives (methyl as represent) are similar with diphenyl-1,3,5-hexatriene (DPH) derivatives while the better conjugation of the thiophen groups attributed to smaller HOMO-LUMO gap, and lower S_1 and T_1 energy levels. Noteworthy, the E(S_1)s are more sensitive to the surrounding polarization with ~0.2 eV red shifted from vacuum to polar solvents, while the E(T_1)s keep almost identical. Shelving the disputes of S_1 configurations, the FMOs dominated T_1 (1^3Bu) of DTHs are about 0.2 eV lower than those of the DPH references. The T_1 energy of DPH has been experimentally determined to be 1.47 eV which indicates the E(T_1) of DTH alkyl derivatives should, with confidence, be around 1.2-1.4 eV.[1]

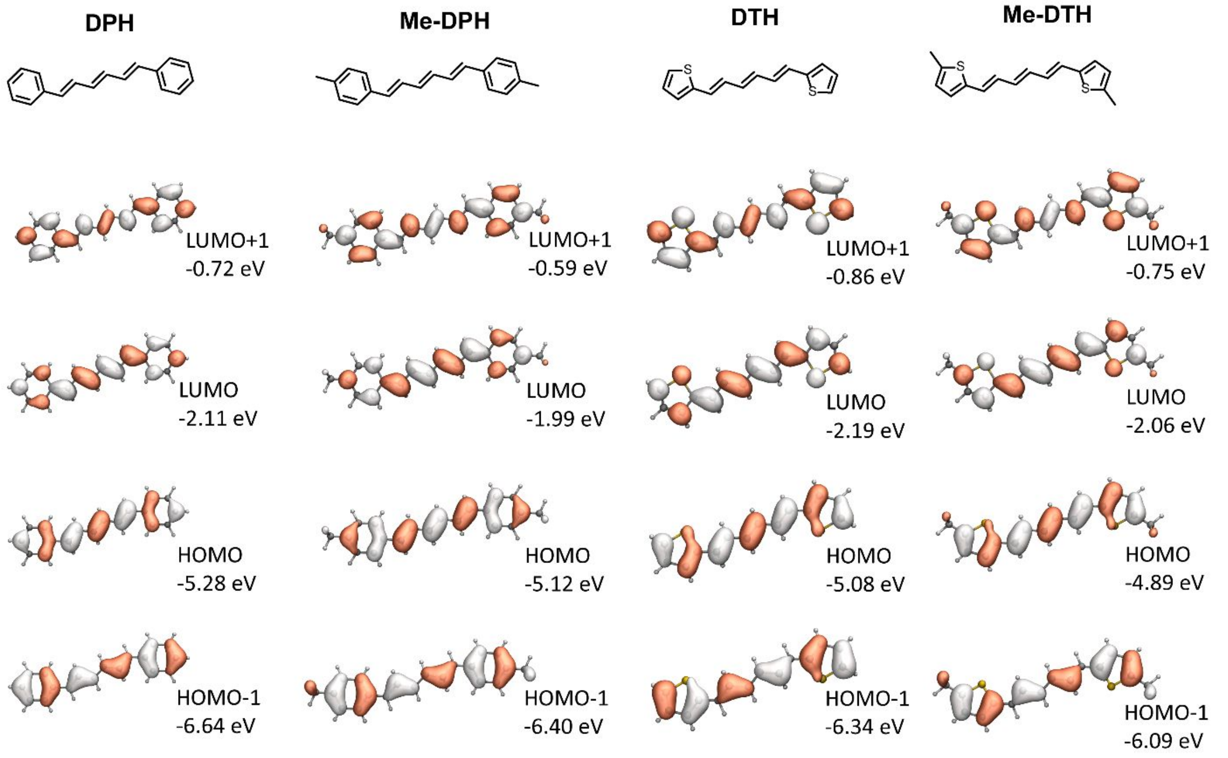

Figure S1 Molecular orbital distributions and relevant energy levels with optimized ground state structures of **DPH**, **Me-DPH**, **DTH** and **Me-DTH** models.

Table S1 Key parameters of the first and second low lying singlet and triplet states of **DPH**, **Me-DPH**, **DTH** and **Me-DTH** models

mol	solvent	n	eV-S[a]	*f*[b]	Orb. Config.	State	eV-T[c]	Orb. Config.	State	T_1v[d]	T_1a[e]
**DPH**	vacuum	1	3.45	2.0394	H-L:0.98;H1-L1:0.024	1^1Bu	1.88	H-L:0.893;H1-L1:0.037	1^3Bu	2.38	1.64
		2	4.71	0	H-L1:0.523;H-L3:0.17	2^1Ag	3.15	H1-L:0.46;H-L1:0.419	1^3Ag		
	toluene	1	3.25	2.1921	H-L:0.976;H1-L1:0.023	1^1Bu	1.88	H-L:0.894;H1-L1:0.037	1^3Bu		
		2	4.64	0	H-L1:0.606;H1-L:0.17	2^1Ag	3.16	H1-L:0.46;H-L1:0.419	1^3Ag		
	chloroform	1	3.27	2.1849	H-L:0.976;H1-L1:0.023	1^1Bu	1.89	H-L:0.894;H1-L1:0.037	1^3Bu		
		2	4.65	0	H-L1:0.607;H1-L:0.151	2^1Ag	3.16	H1-L:0.46;H-L1:0.42	1^3Ag		
**Me-DPH**	vacuum	1	3.40	2.242	H-L:0.977;H1-L1:0.026	1^1Bu	1.86	H-L:0.889;H1-L1:0.038	1^3Bu	2.38	1.63
		2	4.65	0	H-L1:0.332;H-L3:0.303	2^1Ag	3.11	H1-L:0.464;H-L1:0.416	1^3Ag		
	toluene	1	3.21	2.3733	H-L:0.974;H1-L1:0.024	1^1Bu	1.87	H-L:0.889;H1-L1:0.038	1^3Bu		
		2	4.58	0	H-L1:0.398;H1-L:0.356	2^1Ag	3.12	H1-L:0.465;H-L1:0.416	1^3Ag		
	chloroform	1	3.22	2.3688	H-L:0.974;H1-L1:0.024	1^1Bu	1.87	H-L:0.889;H1-L1:0.037	1^3Bu		
		2	4.59	0	H-L1:0.39;H1-L:0.349	2^1Ag	3.12	H1-L:0.466;H-L1:0.415	1^3Ag		
**DTH**	vacuum	1	3.21	1.8196	H-L:0.972;H1-L1:0.032	1^1Bu	1.66	H-L:0.897;H1-L1:0.054	1^3Bu	2.23	1.48
		2	4.43	0	H-L1:0.55;H1-L:0.386	2^1Ag	2.77	H1-L:0.475;H-L1:0.446	1^3Ag		
	toluene	1	3.00	1.9885	H-L:0.971;H1-L1:0.029	1^1Bu	1.66	H-L:0.897;H1-L1:0.054	1^3Bu		
		2	4.28	0	H1-L:0.479;H-L1:0.476	2^1Ag	2.78	H1-L:0.474;H-L1:0.447	1^3Ag		
	chloroform	1	3.02	1.9799	H-L:0.971;H1-L1:0.029	1^1Bu	1.66	H-L:0.897;H1-L1:0.054	1^3Bu		
		2	4.29	0	H-L1:0.479;H1-L:0.475	2^1Ag	2.78	H1-L:0.473;H-L1:0.448	1^3Ag		
**Me-DTH**	vacuum	1	3.13	1.9536	H-L:0.969;H1-L1:0.033	1^1Bu	1.62	H-L:0.893;H1-L1:0.056	1^3Bu	2.20	1.45
		2	4.33	0	H1-L:0.678;H-L1:0.264	2^1Ag	2.70	H1-L:0.483;H-L1:0.44	1^3Ag		
	toluene	1	2.93	2.1076	H-L:0.968;H1-L1:0.03	1^1Bu	1.62	H-L:0.893;H1-L1:0.057	1^3Bu		
		2	4.18	0	H1-L:0.605;H-L1:0.351	2^1Ag	2.70	H1-L:0.483;H-L1:0.44	1^3Ag		
	chloroform	1	2.95	2.1005	H-L:0.968;H1-L1:0.03	1^1Bu	1.62	H-L:0.894;H1-L1:0.057	1^3Bu		
		2	4.19	0	H1-L:0.615;H-L1:0.34	2^1Ag	2.70	H1-L:0.483;H-L1:0.44	1^3Ag		

[a]Vertical excitation energy of singlet on TD-DFT M06-2X/def2-TZVP. [b]Oscillator strength. [c]Oscillator strength. [c]Vertical excitation energy of triplet on TD-DFT M06-2X/def2-TZVP. [d]Verticle and [e]adiabatic low lying triplet energy on ΔSCF DFT M06-2X/def2-SVP

S3 Single Crystal X-ray Diffraction

Single-crystal X-ray diffraction data were collected on a Bruker D8-QUEST diffractometer, equipped with an Incoatec I□S Cu microsource (□ = 1.5418 Å) and a PHOTON-III detector operating in shutterless mode. The temperature was controlled by an Oxford Cryosystems open-flow N_2 Cryostream operating at 180(2) K. The control and processing software was Bruker *APEX*4. The diffraction images were integrated using *SAINT* in *APEX*4, and a multi-scan correction was applied using *SADABS*. The final unit-cell parameters were refined against all reflections over the full data range. The structure was solved using *SHELXT* and refined using *SHELXL*.[2,3]

	**Hex_DTH**	**EH_DTH**	**OB_DTH**
CCDC number	2179619	2179618	2179620
Cambridge data number	HB_B1_0038	HB_B2_0037	HB_B1_0039
Chemical formula	$C_{26}H_{36}S_2$	$C_{30}H_{44}S_2$	$C_{38}H_{60}S_2$
Formula weight	412.67	468.77	580.98
Temperature / K	180(2)	180(2)	180(2)
Crystal system	monoclinic	monoclinic	monoclinic
Space group	C2/c	$P2_1/c$	$P2_1/n$
a / Å	15.1860(6)	19.940(7)	11.8297(8)
b / Å	5.6905(2)	5.8996(15)	5.5589(3)
c / Å	28.3093(12)	12.666(4)	27.6264(18)
alpha / °	90	90	90
beta / °	104.5690(10)	104.66(2)	98.647(4)
gamma / °	90	90	90
Unit-cell volume / $Å^3$	2367.71(16)	1441.5(8)	1796.1(2)
Z	4	2	2
Calc. density / g cm^{-3}	1.158	1.080	1.074
F(000)	896	512	640
Radiation type	CuK□	CuK□	CuK□
Absorption coefficient / mm^{-1}	2.078	1.757	1.492
Crystal size / mm^3	0.24 x 0.18 x 0.12	0.18 x 0.14 x 0.02	0.35 x 0.04 x 0.02
2-Theta range / °	15.05-133.10	4.58-109.69	6.47-134.04
Completeness to max 2□	0.991	0.978	0.991
No. of reflections measured	22937	11330	29392
No. of independent reflections	2084	1760	3185
R(int)	0.0275	0.1833	0.1595
No. parameters / restraints	128 / 0	194 / 152	204 / 24
Final R1 values (I > 2□(I))	0.0253	0.1131	0.0668
Final $wR(F^2)$ values (all data)	0.0687	0.3327	0.1906
Goodness-of-fit on F^2	1.046	1.107	1.086

Largest difference peak & hole / e Å^{-3}	0.192, -0.219	0.552, -0.290	0.720, -0.348

Hex-DTH

Crystals were relatively large and block-like, and produced strong diffraction. Structure refinement was correspondingly straightforward. The molecule is situated on a crystallographic inversion centre in space group C2/c. The DTH core is planar and the hexyl chains are fully extended with no evident disorder. The DTH units form offset stacks along the b axis and the stacks of molecules are arranged in a brick-work pattern so that alkyl chains lie across DTH faces in neighbouring molecules and eliminate direct interaction between DTH units in different columns.

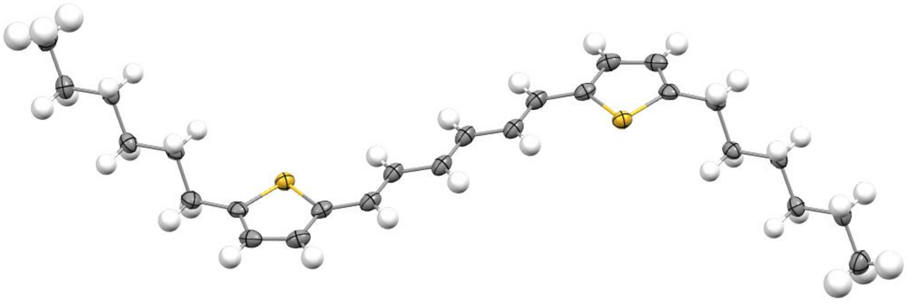

Figure S2: Molecular structure in Hex-DTH, showing displacement ellipsoids at 50% probability.

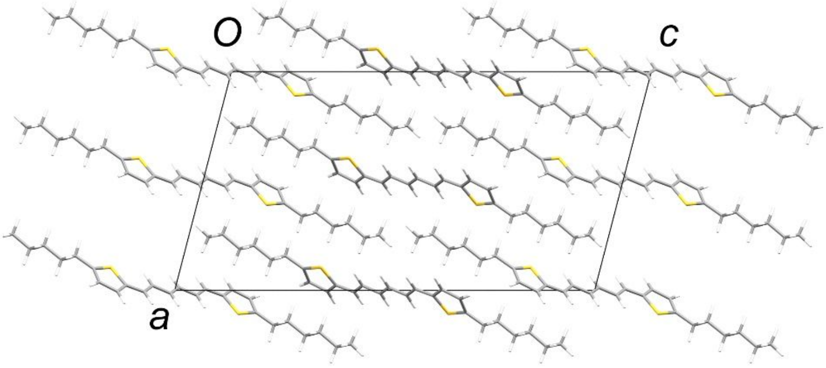

Figure S3: Crystal packing in Hex-DTH.

OB-DTH

Crystals were thin plates (min dimension 0.02 mm), which produced relatively weak diffraction at higher angles. Structure solution and refinement was largely straightforward, although disorder was evident in the alkyl chains. The molecule is situated on a crystallographic inversion centre in space group $P2_1/n$, with the DTH core close to planar, but showing a minor undulation. The alkyl chains are modelled as two disorder components: (1) the "major" component (ca 60%, shown in standard element colours in the diagram) shows a fully extended octyl chain, with a fully extended butyl branch; (2) the "minor" component (ca 40%, shown in red in the diagram) exchanges the positions of the two chains, and the longer chain is bent. The 1,1- and 1,2- distances along the chains were restrained to retain sensible chemical geometry, and it was possible to refine all C atoms with

unrestrained anisotropic displacement parameters (*i.e.* the disorder was quite clearly resolved). The chains in neighbouring molecules meet to produce a rectangular arrangement and the two disorder components represent different ways in which the chains can fit within this arrangement. As in Hex-DTH, the DTH cores are arranged into offset 1-D stacks. The geometry of the stacks is approximately identical in OB-DTH and Hex-DTH. The stacks are again arranged into a brick-work structure where the alkyl chains lie close to the DTH faces and eliminate direct interaction between DTH units in different columns.

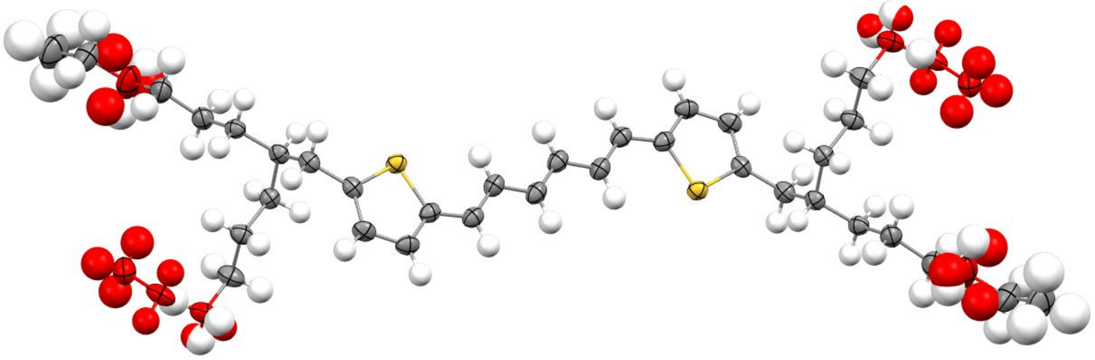

Figure S4: Molecular structure in OB-DTH, showing displacement ellipsoids at 50% probability. The atoms coloured red comprise disorder component (2).

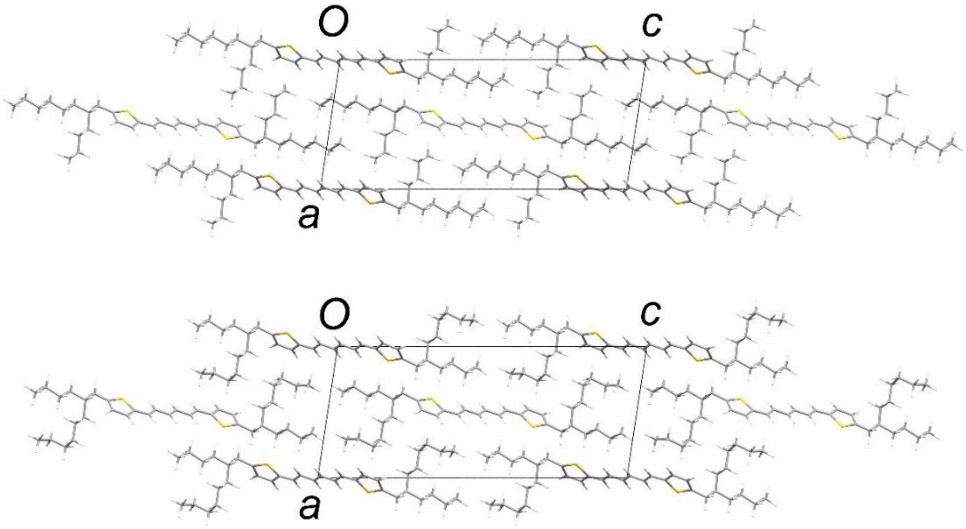

Figure S5: alternative arrangements of alkyl chains within the OB-DTH crystal structure.

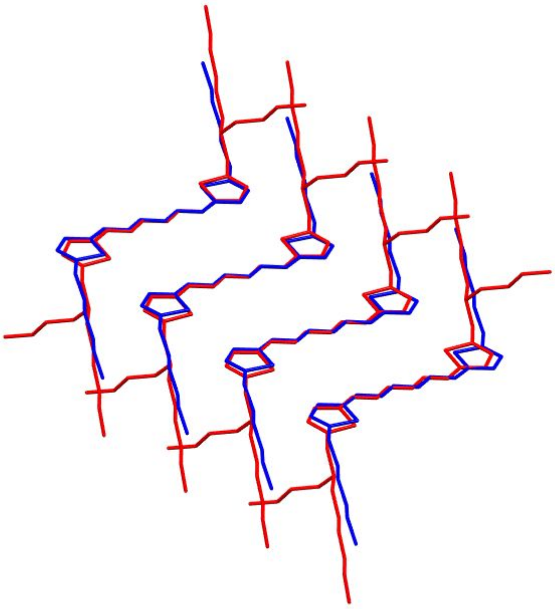

Figure S6: Offset stacks in Hex-DTH (blue) and OB-DTH (red). The relative positions of the DTH cores are approximately identical in the two structures.

<u>EH-DTH</u>

Structure determination for EH-DTH was challenging. Many crystals from numerous batches were analysed and the presented data is the best obtained. Crystals were thin plates (typical min dimension 0.02 mm), which produced weak diffraction at higher angles. The diffraction patterns consistently showed broadened and split peaks, which is not uncommon for lamellar-type structures. Representative reconstructed precession images are as follows:

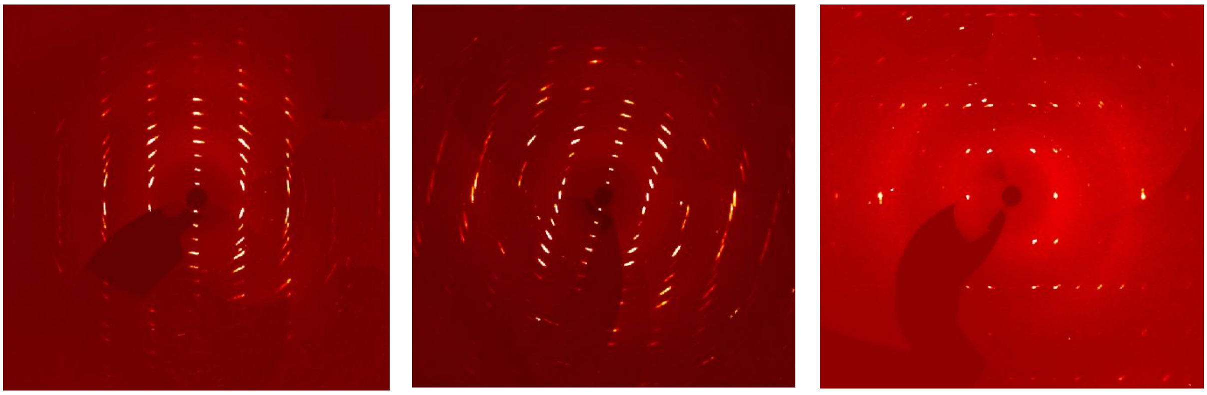

0kl **h0l** **hk0**

The reported data were integrated as a single domain. For the resulting data set, I/□(I) falls below 3.0 around 1.0 Å. The data were truncated to 0.95 Å, with mean I/□(I) ≈ 1.5 for the outer shell. This produced an overall R(int) ≈ 18%, with *ca* 60% of the unique data observed at the 3□ level.

During structure solution, the DTH core was immediately clear, situated on a crystallographic inversion centre in space group $P2_1/c$. The alkyl chains were harder to identify and showed disorder. The chain is modelled as two disorder components in an approximately 50:50 ratio. One component

shows a fully extended butyl chain, while the other is bent at the terminal CH_2–CH_3 bond. The ethyl branch is comparable in the two cases. Due to the inversion symmetry imposed on the molecule by the choice of space group $P2_1/c$, the two disorder components are overlaid in the asymmetric unit (comprising half of the molecule). The 1,1- and 1,2- distances along the chains were restrained to retain sensible chemical geometry, and the C atoms were refined with anisotropic displacement parameters restrained to resemble isotropic behaviour. Post-refinement analysis of the data set (using TWINROTMAT in PLATON[4]) did not identify any twinning model that may improve the refinement.

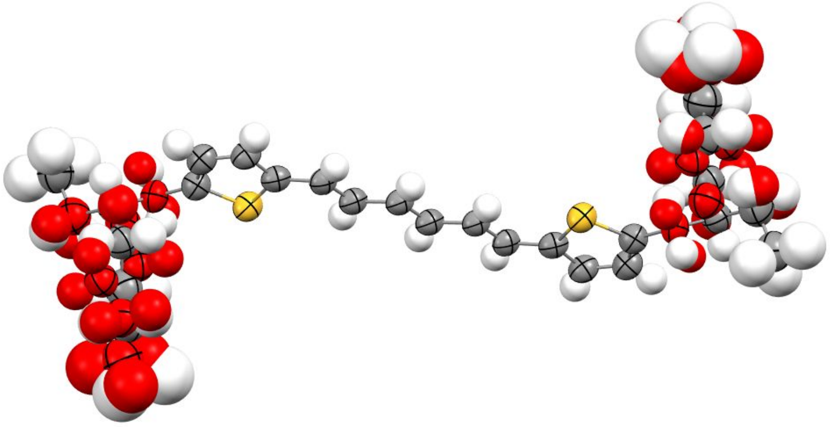

Figure S7: Molecular structure in EH-DTH, showing displacement ellipsoids at 50% probability. The atoms coloured red comprise disorder component (2).

In contrast to Hex-DTH and OB-DTH, the molecules in EH-DTH are arranged clearly into layers parallel to the bc planes of the unit cell, with the DTH cores and alkyl chains segregated into alternating planar regions. The DTH cores are aligned in a herring-bone manner, with an angle of ca 50° between neighbouring core planes. The arrangement is similar to that in unsubstituted DTH (CSD refcode: TDTHTR01), although the molecules in **EH–DTH** are shifted laterally relative to each other and spaced more widely to accommodate the packing requirements of the alkyl chains.
In the alkyl chain regions, some unreasonably short intermolecular contacts exist between chains belonging to the same disorder component, i.e. a molecule adopting one disorder component cannot be next to a molecule adopting the same disorder component. To produce a model that eliminates these short contacts, the symmetry must be reduced by removing the inversion centre within the molecule (yielding space group Pc). Retaining one disorder component at one end of the molecule and the other disorder component at the other end produces a physically reasonable model in which all alkyl regions are identical by translation (but not by inversion).

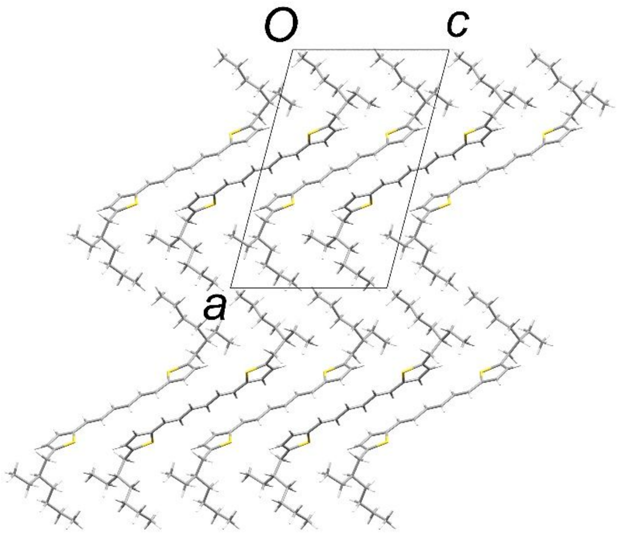

Figure S8: Crystal packing in EH-DTH, showing molecules segregated into alternating DTH core and alkyl chain layers.

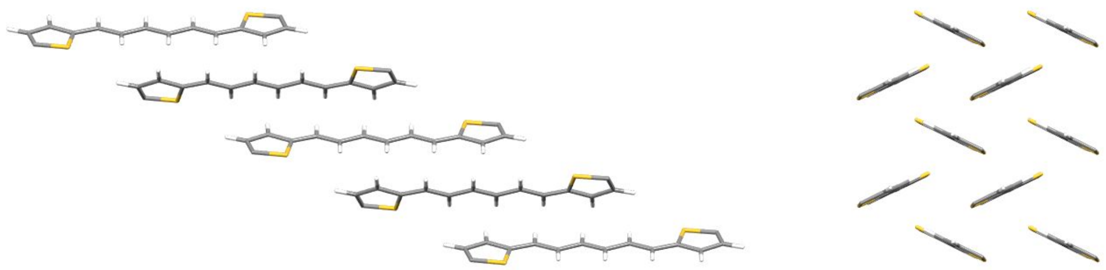

Figure S9: Perpendicular views of the edge-to-face interactions between DTH cores within the lamellar structure of EH-DTH (alkyl chains omitted).

Structure validation using periodic dispersion-corrected DFT calculations

As an additional validation step, the crystal structures of Hex-DTH, OB-DTH and EH-DTH were optimised using periodic dispersion-corrected DFT calculations, using CASTEP[5] via the interface in Materials Studio. The PBE exchange-correlation functional was applied[6], with a dispersion correction according to Grimme[7]. The plane-wave basis-set cut-off was set to 310 eV and all other parameters were set to the "Fine" defaults in Materials Studio. Unit-cell parameters were constrained in each case to those from the reported crystal structure. Prior to energy minimisation, the positions of H atoms in the X-ray structures were normalised using the default settings in Mercury to produce starting positions close to nuclear positions. For OB-DTH, two models (both in space group $P2_1/n$) showing the alternative arrangements of the alkyl chains were optimised separately. For EH-DTH, the symmetry of the structure was reduced to Pc to eliminate the alkyl chain disorder, as described in the preceding section. Minimised structures were compared to the starting structures using the method described by van de Streek and Neumann.[8] The two disordered structures naturally result in

larger displacements relative to the non-disordered Hex-DTH. The larger H-atom displacements in OB-DTH and EH-DTH are associated with the disordered alkyl chains, particularly rotations of the terminal CH_3 groups relative to the calculated positions applied in the X-ray structures. The non-H RMS displacements of 0.110 Å or less are well below the benchmark upper limit of 0.25 Å established for optimisation of correct molecular crystal structures. The DFT-D results add confidence to the correctness of the structures that show less than optimal crystallographic indicators, and they also yield a clearer picture of the likely local arrangements of the alkyl chains in the disordered cases.

Cartesian displacement (Å)	**Hex-DTH**	**OB-DTH: part (1)**	**OB-DTH: part (2)**	**EH-DTH**
All atom RMS	0.044	0.121	0.173	0.177
All atom maximum	0.086 [H13A]	0.375 [H15C]	0.747 [H13C]	0.446 [H11D]
**Non-H RMS**	**0.027**	**0.076**	**0.074**	**0.110**
Non-H maximum	0.052 [C5]	0.151 [C18]	0.174 [C21]	0.255 [C10A]

S4 Grazing Incidence Small Angle X-ray Scattering

Grazing incidence small- and wide-angle X-ray scattering (GISAXS/GIWAXS) was performed on the Xuess instrument equipped with an Excillum MetalJet liquid gallium X-ray source. Alignment was performed on silicon substrates via three iterative height (z) and rocking curve (Ω) scans, with the final grazing incidence angle set to Ω = 0.2°. Scattering patterns were recorded on a vertically-offset Pilatus 1M detector with a sample to detector distance of 352 mm, calibrated using a silver behenate standard to achieve a *q*-range of 0.045 – 1.85 $Å^{-1}$. Two-dimensional images were recorded with exposure times of 900 s. The images were masked to remove the sample horizon, detector module gaps and beamstop and radially integrated from the apparent beam centre. Data correction and reduction was performed using the GIXSGUI MATLAB toolbox.[9] Two-dimensional scattering data was reduced to one-dimensional via radial integration, which was performed with a mask to remove contributions from "hot pixels", the substrate horizon and reflected beam.. For GIWAXS measurements, films of Hex–DTH, EH–DTH, & OB–DTH were deposited on plasma cleaned silicon substrates (20 x 20 mm) via spin-coating. Film deposition was performed inside a nitrogen filled glovebox, with 50 μL of either Hex–DTH, EH–DTH, & OB–DTH in chloroform (15 mg/ml) deposited on the silicon substrate, which was then spun at 1,500 rpm for 120s. The film samples were stored inside the glovebox overnight to allow the solvent to fully evaporate.

S5 Transient Absorption Spectroscopy

Sensitization of EH-DTH

A dilute solution of EH-DTH at 0.5mg/ml was prepared with 10%mol PDOEP, excited at 532 nm to selectively excite PDEOP, which then sensitizes the EH-DTH, revealing the triplet PIA.
We will also use this measurement to calculate the triplet cross section in terms of dT/T and further to calculate the number of triplets per unit area in various DTHs. Below are the figures showing area normalised spectra of PDOEP Solution in Toluene and 0.5 mg/ml EH-DTH Solution with 10% mol PDOEP (Sensitized EH-DTH).

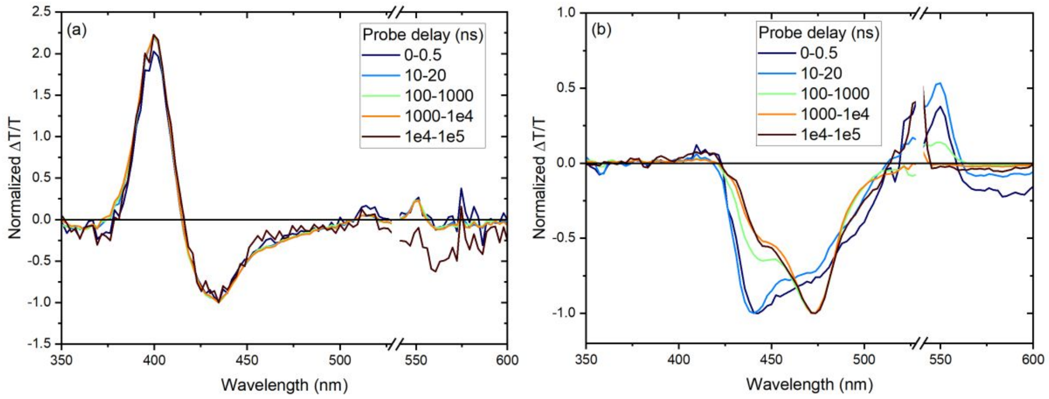

Figure S10 – Figure **A)** shows the normalised Pump Probe Spectra of PDOEP solution excited at 532 nm while Figure **B**) shows the normalised Pump Probe Spectra of the Sensitized Solution excited at 532 nm to selectively excite PDOEP. The peak around 475 nm in figure B is from the triplet PIA of EH-DTH.

Below we will show the figure with normalised kinetics of PDOEP GSB in both solutions at PDOEP GSB peak position as well as at EH-DTH Peak Position and kinetics of the sensitized solution. We can see a decline in PDOEP GSB with a corresponding rise in EH-DTH triplet PIA. Note- the position of the triplet PIA is also in excellent agreement with the PIA feature that appears in concentrated solutions and is not present in dilute solutions of EH-DTH, as seen in figure **S12,** further confirming the correct assignment of this feature as EH-DTH Triplet PIA.

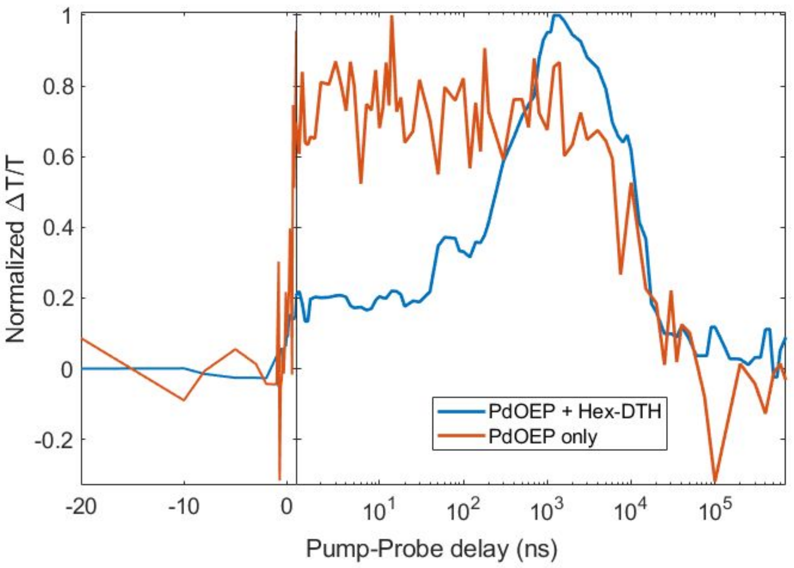

Figure S11 – This figure shows kinetics of PIA at 475 nm in both solutions of PDOEP and the sensitized Solution. It reveals the rise of the triplet PIA peak in the sensitized solution over a period of microseconds. Moreover we can use the strengths of the signal to find out the triplet cross section as we will do in the section below.

Calculation of Singlet Fission Yield

To calculate the singlet fission yield, we require singlet and triplet exciton populations. We can use the optical density at the wavelength of the laser and incoming power of the laser to ascertain the number of singlets excitons produced. However, a couple more steps are required to calculate the number of triplet excitons, as they are not produced through direct absorption of energy from the ground state and they do not decay radiatively to the ground state. Their dipole allowed transitions to higher lying triplet states, appearing in the form of a PIA signal in the transient absorption spectra can help quantify their populations. If the absorption cross section ($\boldsymbol{\sigma_T}$) is known, the triplet population can be deduced from strength of the triplet PIA, from the following equation $dt/T = n_T\boldsymbol{\sigma_T}$**,** where n_T is the average density of triplets per unit area and dt/T is the fractional change in transmission caused by the triplet PIA.[10]

We use the sensitized solution to estimate the cross section, with the following assumptions –

a) All the singlets formed in PDOEP inter system cross to triplets. This assumption is justified as PDOEP is known to intersystem cross with near unity efficiency.

b) The dominant decay pathway is transfer of triplet energy to DEH-DTH molecules. This can be seen as a decay of PDOEP ground state corresponds nearly perfectly with the rise of the DEH-DTH triplet PIA.

c) Triplet cross section remains the same in all three materials as they have the same moiety and different side chains.

The number of singlets can be found from the laser fluence and power absorbed assuming no scattering losses as is expected in these low concentration solutions, and taking into account reflection losses.

Since we can ascertain the number of singlets, we can ascertain the number of triplets in PDOEP and furthermore looking at the decay of the ground state bleach, we are able to ascertain the transfer efficiency and thereby the number of DEH-DTH triplets.

Calculation of DTHs` Triplet Cross Section From Sensitization Data.

After this we calculate the area density of triplets using the laser spot size and then deduce σ_T *from the sensitization TA data.*

dt/T = $n_T\sigma_T$, **(1)**

This implies $\sigma_T = (dt/T)/n_T$

ΔT */T contribution from EH-DTH Triplet PIA* = -60E-03

$n_{T\,(EH)} = n_{T\,(PDOEP)}$ ***Efficiency of Transfer** ***(2)***

Efficiency of Transfer = (rate of rise of Triplet PIA of Hex-DTH/ (rate of rise of Triplet PIA of Hex-DTH + rate of decay of triplet PIA of PdOEP triplet)) *100% = 99% (3)

$n_{T(PDOEP)} = n_{S(PDOEP)}$ = ***Fluence/Energy of Each Photon*** **= 1.54732E+13 (4)**

$n_{T(EH)}$ = 1.54732E+13*99% = 1.53185E+13

$\sigma_{T(EH)}$ = -9.3E-16 cm^{-2} (5)

Using (5) and assumption c) $\sigma_{TEH)} = \sigma_{T(OB)} = \sigma_{T(H)}$

Calculation of Singlet Fission Yield in Films

The three films of DTHs were excited with 355 nm laser to generate S1 excitons.
SF Yield = n_{T1}/n_{S1}

For n_{S1},we use fluence and film ODs accounting for reflection losses to deduce the number of singlets generated and thereby the number of singlets per unit area n_{S1} using equation (4) as above.

For n_{T1} we use σ_T and dt/T to deduce *the area density of triplets (n_T) using equation (1) and value of dt/T from the data and **σ_T from above equation (5)**.*

This gives us the following yields
1.17E+02 Hex-DTH
8.1E+01 EH-DTH
1.93E+02 OB-DTH

Error Calculation

Error in triplet cross section (in terms of dt/T peak) From Sensitization

$\Delta\, \sigma_{Tsolution} / \sigma_{Tsolution} = ((\Delta\, (dt/T)/(dt/T))^2 + (\Delta\, n_{Tsolution}/ n_{Tsolution})^2)^{1/2}$

$\Delta\, n_{Tsolution}/ n_{Tsolution}$ = $\Delta\, n_{SPDOEP}/ n_{SPDEOP}$ = Δ Fluence/ Fluence = Δ Power Absorbed/ Power Absorbed = 1/50 = 0.02

(Δ (dt/T)/(dt/T))= 0.02 from signal to noise ratio of the setup

$\Delta\, \sigma_{Tsolution} / \sigma_{Tsolution} = 0.028$

Error in yield

Δ Yield/ Yield $= ((\Delta\, n_{Sfilm}/ n_{Sfilm})^2 + (\Delta\, n_{Tfilm}/ n_{Tfilm})^2)^{1/2}$ (1)

For Hex-DTH = $\Delta\, n_S/ n_S = 1/12 = 0.083$ (2)
For EH-DTH = $\Delta\, n_S/ n_S = 1/15 = 0.067$ (3)
For OB-DTH = $\Delta\, n_S/ n_S = 1/12 = 0.083$ (4)

$n_{Tfilm} = (dt/T)/ \sigma_{Tsolution}$

$\Delta\, n_{Tfilm}/ n_{Tfilm} = ((\Delta\, (dt/T)/(dt/T))^2 + (\Delta\, \sigma_{Tsolution} / \sigma_{Tsolution})^2)^{1/2}$

$\Delta\, (dt/T)/(dt/T) = 0.02$
$\Delta\, \sigma_{Tsolution} / \sigma_{Tsolution} = 0.028$

so $\Delta\, n_{Tfilm}/ n_{Tfilm} = 0.035$

Using (1, 2, 3, 4) we get

Δ Yield/ Yield = 0.090, 0.076, 0.090 for Hex-DTH, EH-DTH and OB-DTH

Solution Concentration Series of DEH-DTH

Solutions of EH-DTH were made at various concentrations (0.5 mg/ml, 5mg/ml and 50 mg/ml). This acts as a great litmus test for singlet fission as we expect singlet fission to be turned on at higher concentrations of solution as intermolecular interaction (between a molecule with S1 excited state and a molecule in the ground state) being key for singlet fission.

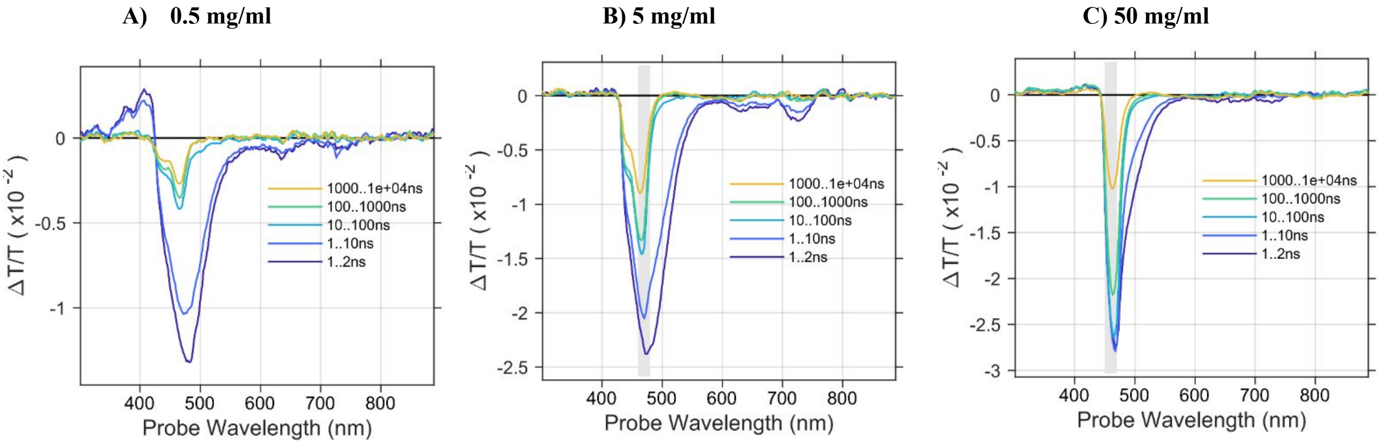

Figure S12 – The figure shows a sharp feature appearing as we increase the concentration- we assign this to the triplet PIA. It is also in excellent agreement with the feature in sensitized EH-DTH solution.

Pump Probe Spectroscopy of DTH Films

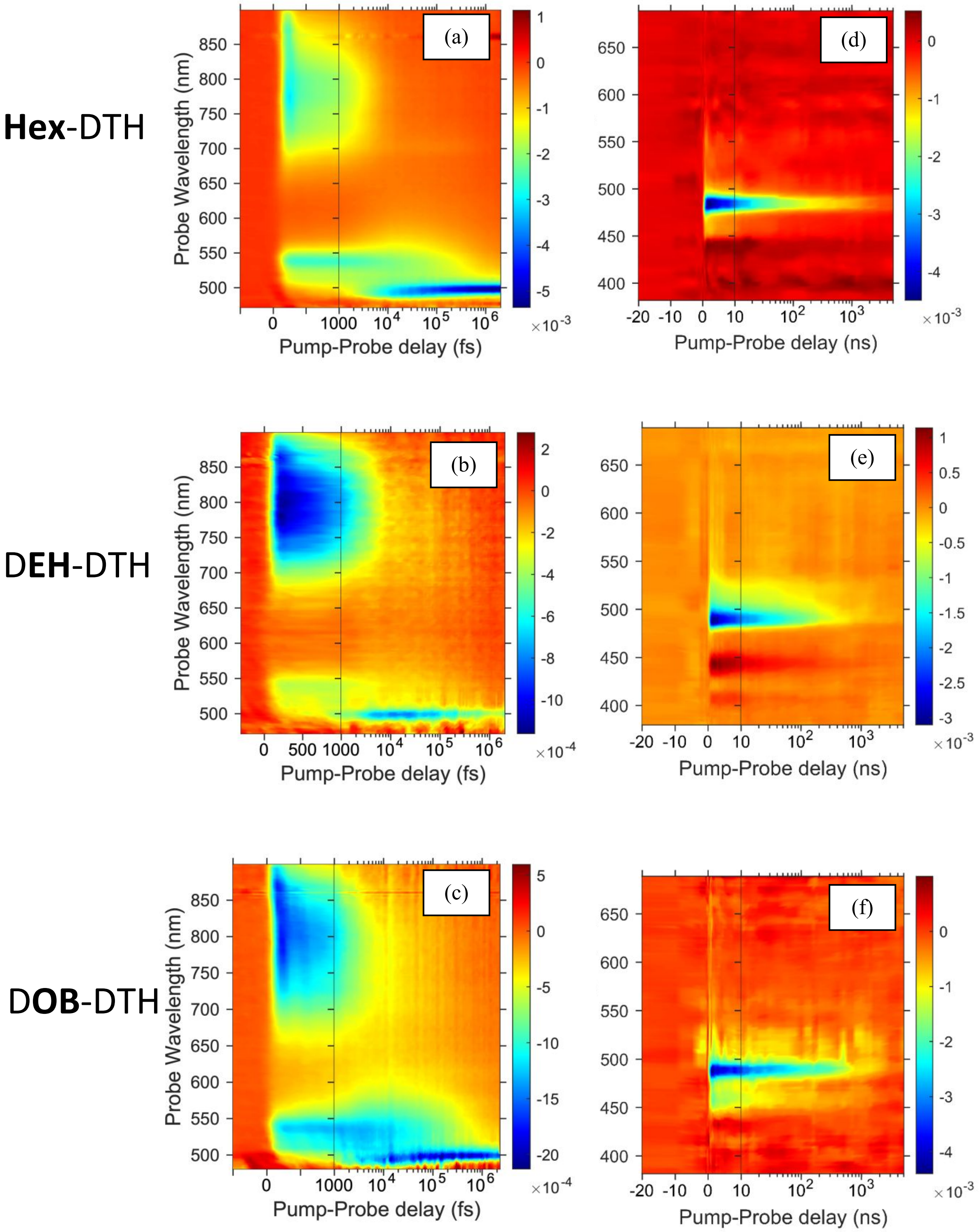

Figure S13 : 2D Maps of Pump Probe Transient Absorption Spectrum Taken At Different Timescales -500 to 2M femtoseconds for (a) Hex-DTH, (b) EH-DTH and (c) OB-DTH, and at -20 to 10K nanoseconds for (d) Hex-DTH, (e) EH-DTH and (f) OB-DTH.

Figure S13 (a), (b) and (c) show the transition of the singlet with the broad PIA at 500-550 nm and 700- 900 nm to an intermediate state with a red shifted PIA through the first 10 picoseconds, to a final state at 450-500 nm which starts to appear strongly within 100 ps in all the systems, which we will later assign as the triplet state. Normalising using the singlet signature, the strongest and cleanest triplet PIA signature is in the Di Hexyl DTH films followed by the Di Octyl Butyl DTH films, the weakest being in the DEH-DTH films, while the longest lived singlet-TT state signatures and transitions are in the Di Ethyl Hexyl DTH films followed by the Di Octyl Butyl DTH films while the fastest and short lived transitions occurs in the Di- Hexyl DTH film.

In this initial study, we show singlet fission occurring efficiently in these new materials. We will not probe further into the science behind the differences in photo physics between these three materials. However the data in figures S13 a), b) and c) clearly shows that Hex-DTH undergoes the fastest transitions.

Figure S13 (d), (e) and (f) show the triplets assigned to the 480-490 nm signature and singlet related stimulated emission feature assigned to positive signature at 430-460 nm.

A) Hex-DTH **B) EH-DTH** **C) OB-DTH**

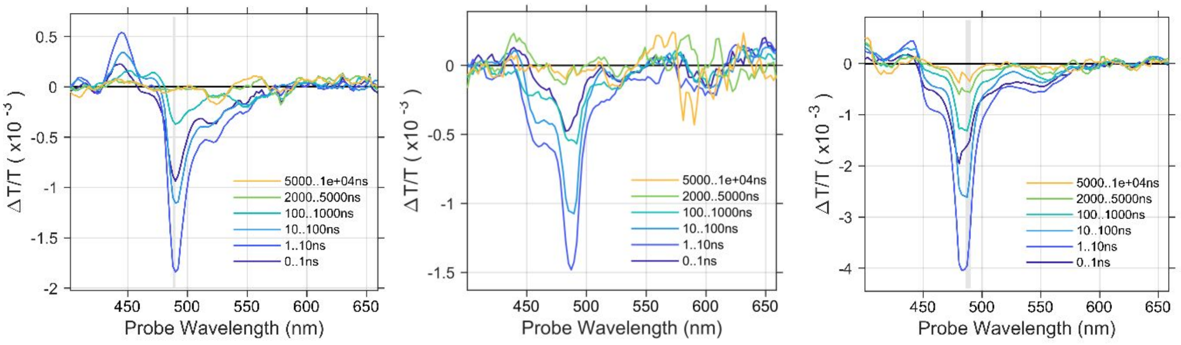

Figure S14 – This figure shows pump probe spectra at various time points for A) Hex-DTH, B) EH-DTH and C) OB- DTH films. One can see an initial rise of the triplet signal centred around 480-490 nm, within the first 10 nanoseconds and subsequent decay by 1000 ns in all films

OD-DTH film mentioned above in main text, measured in short time using pump probe spectroscopy (femto to picosecond timescale) is given below. The film however was not very stable in long time (nanosecond timescale) due to increased fluence in this setup and low light stability of film.

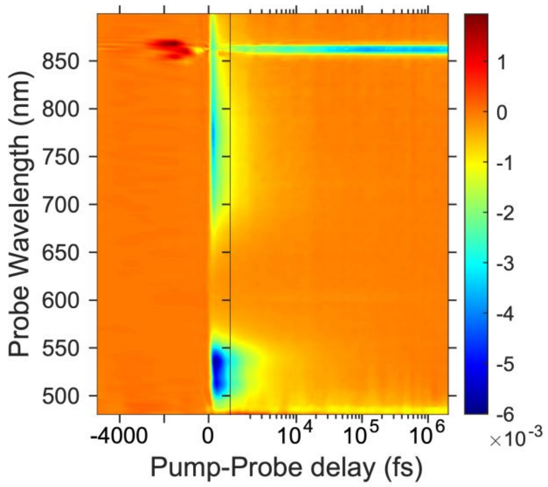

Figure S15 – This figure shows 2D map with pump probe spectra at various time points for OD-DTH films. However since this film was not very stable, we were not able to investigate it further. The signal at 860 nm arises as a doubling of pump scatter (at 430nm pump) which increases as we measure the sample due to degradation, thereby increasing in intensity with increase in pump-probe delay (and time of exposure)

Film Studied	DH	DEH	DOB
Fluence (J/cm^2)	4.68E-06	5.12E-06	5.8-06

Table S2 Excitation fluences used for measuring the transient absorption spectra of different DTH films reported in this manuscript.

Film Preparation

Hex–DTH, EH–DTH, & OB–DTH in chloroform (15 mg/ml) deposited on the glass substrate, which was then spun at 1,500 rpm for 120s

S6 Analysis of electronic interactions in single crystal structure.

Dimer geometries are extracted from experimental single crystals with the protons further optimized at B3LYP/def2-SVP level the Gaussian 16 program. For the orbital energy analysis, the side chains were simplified to methyl group to reduce computation costs. Interaction region indicator (IRI) and charge decomposition analysis (CDA) were performed by Multiwfn 3.8 program according to the program manual and literature method. Key values were collected as **Figure S16-18.**
To further investigate the π-π interaction in the Hex-DTH and OB-DTH crystal, we performed he interaction region indicator (IRI) real space function that reveals weak interactions between the DTH cores. More continuous isosurface in the Hex-DTH dimer indicated slightly stronger Van de Waals interactions comparing with that of the OB-DTH dimer, which could be arising from a subtle nuance of the slip stack angle of the packing motifs. Nevertheless, the long distance and the weak interactions can't afford efficient coupling of the electrons. This can be supported by the charge decomposition analysis (CDA), in which the frontier MOs of the monomers were simply summed to the dimer without obvious interaction or mixture.

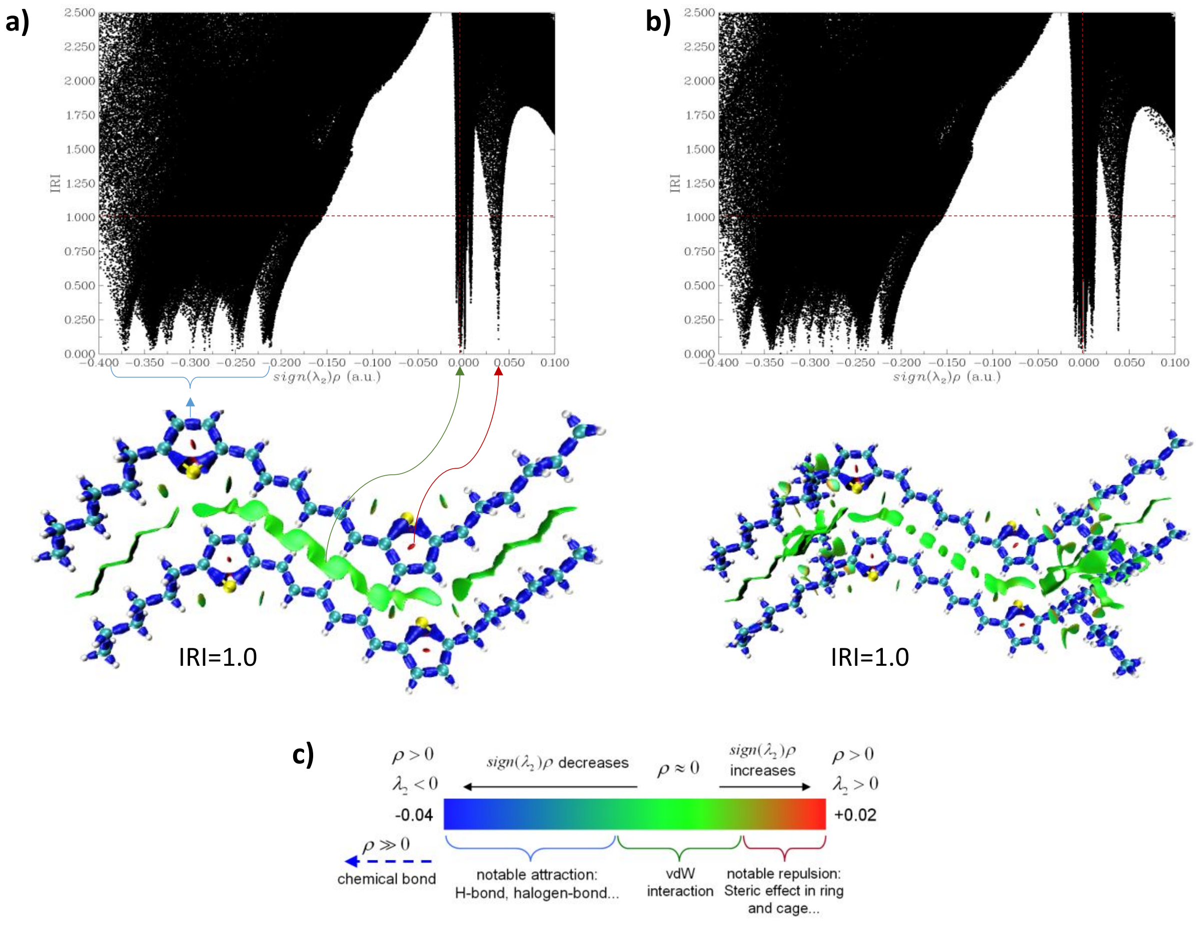

Figure S16. Scatter map between IRI and *sign(λ₂)ρ* (top) and isosurface map of IRI=1.0 (bottom) for (a) **Hex-DTH** and (b) **OB-DTH**. (c) Standard coloring method and chemical explanation of *sign(λ2)ρ* on IRI isosurfaces.

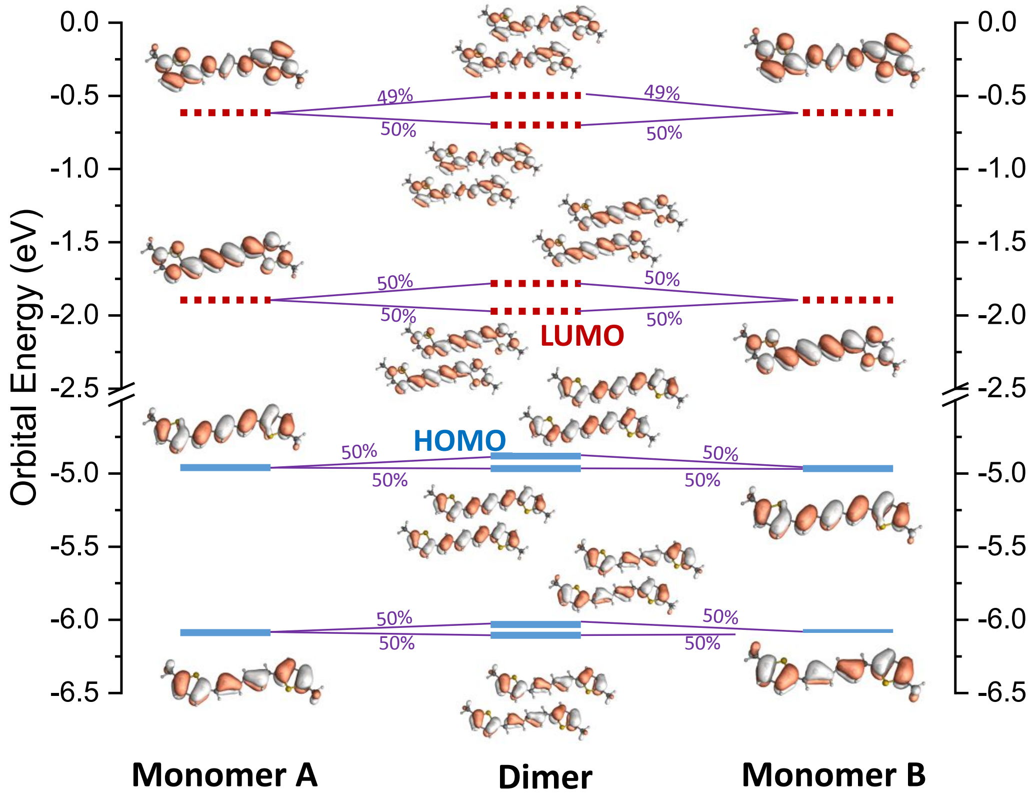

Figure S17 Orbital interaction diagram of **Hex-DTH** dimer. Blue solid and red dashed bars correspond to occupied and unoccupied MOs, respectively. Purple texts indicate major contribution of MOs from the monomers to the MOs of dimer. The orbital compositions were evaluated by Mulliken method.

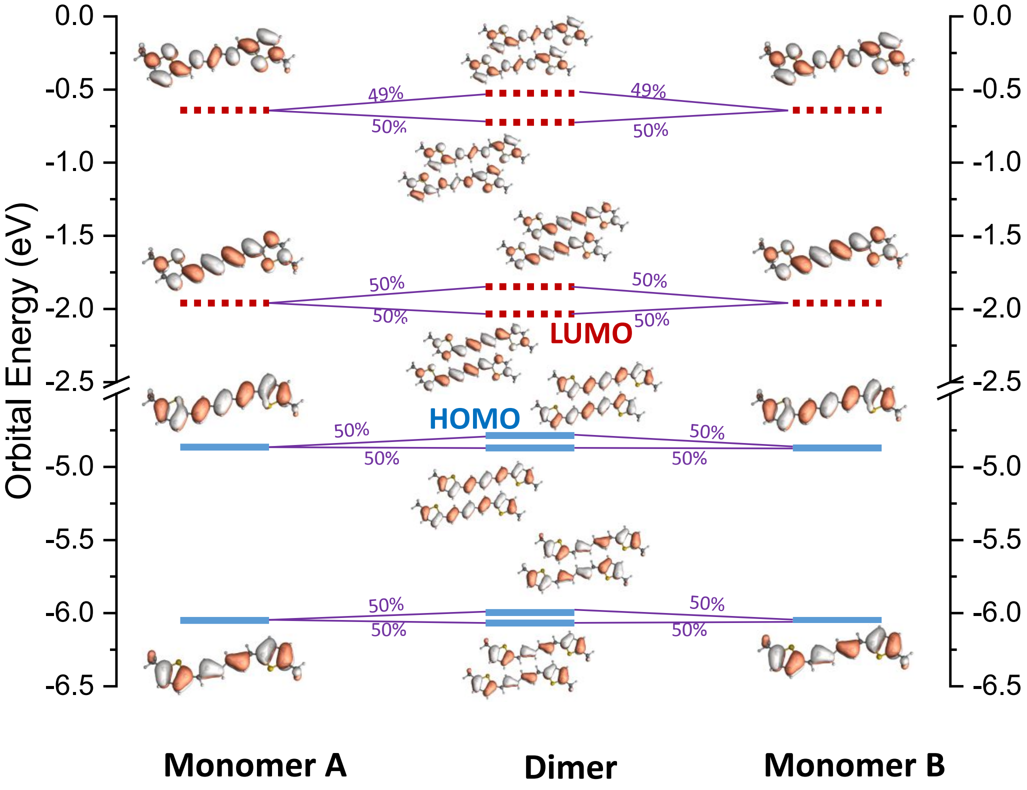

Figure S18 Orbital interaction diagram of **OB-DTH** dimer. Blue solid and red dashed bars correspond to occupied and unoccupied MOs, respectively. Purple texts indicate major contribution of MOs from the monomers to the MOs of dimer. The orbital compositions were evaluated by Mulliken method.

TD-DFT Analysis of transitions within dimers in single crystal geometry

Ground state geometry of the monomer is optimized at B3LYP/def2-SVP level. The dispersion correction was conducted by Grimme's D3 version by the Gaussian 16 program. Dimer geometries are extracted from experimental single crystals with the protons further optimized at the same level for the monomer, the side chains were simplified to methyl group to reduce computation costs. Basing on the above geometries, the vertical excitation energies were evaluated at M06-2X/def2-TZVP by TD-DFT method with polarizable continuum model (PCM) taking the dielectric constant for toluene ($\varepsilon = 2.37$) and chloroform ($\varepsilon = 4.71$) as reference. Excited states analysis was processed with the TDDFT results using Multiwfn 3.8 program according to the program manual and literature method.

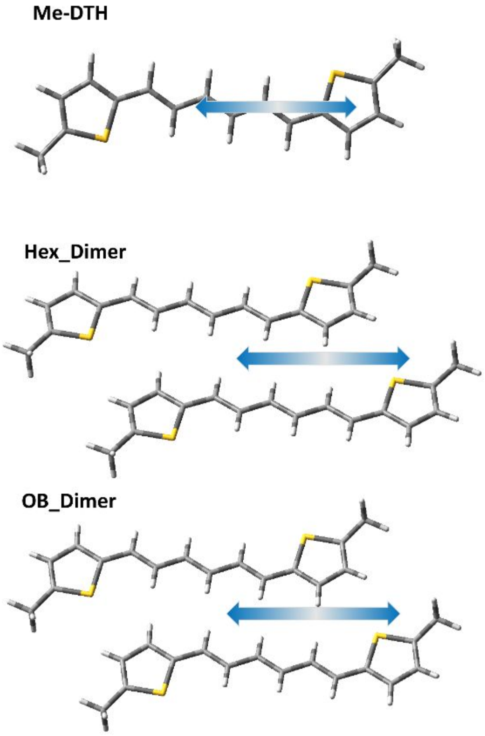

	EX. STATE	EENERGY /EV	OSCILLATOR STRENGTH	ORB. CONFIG.
**Me-DTH Monomer**	S_1	2.933	2.108	H-L: 96.8%
**Hex_Dimer**	S_1	3.164	0	H1-L:58.4%; H-L1:38.2%
	S_2	3.220	3.773	H-L:75.2%;H1-L1:21.6%
**OB_Dimer**	S_1	2.985	0	H1-L:55.9%; H-L1:41.1%
	S_2	3.065	3.794	H-L:75.2%;H1-L1:22.0%

Figure S19 TDM vectors for S_1 of **Me-DTH** Monomer, and S_2 for the dimers extracted from single crystal with different side chains (**Hex** and **OB**).

Figure S19 shows the effect of the different stacking distances on the lowest optical transitions. In the Hex-Dimer the first optically bright state has been considerably blue-shifted relative to the monomer (2.93 eV to 3.16 eV) suggestive of some kind of H-like aggregation. In contrast, in the OB-Dimer the first optically bright transition is almost at the same energy as the monomeric compound. Interestingly, both transitions are almost identical in-terms of their orbital contributions.

S7 Stability

To assess the ambient stability of the new materials Hex-DTH thin films were prepared and exposed to ambient conditions in either the light or the dark. The films appear stable in the dark but degrade over the course of 5 days in the light. The degradation appears to be comparable to that of Tips-Pentacene.[11]

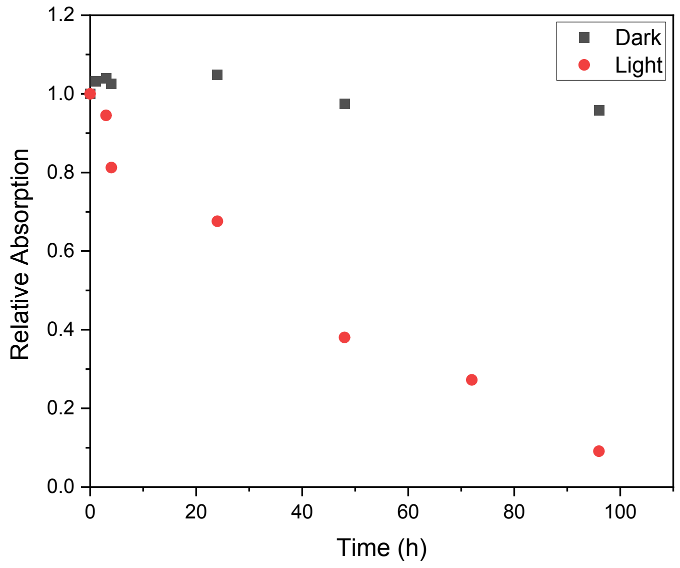

Figure S20 Maximum absorption decrease of Hex-DTH films spun cast from chloroform (7mg/mL) under different conditions.

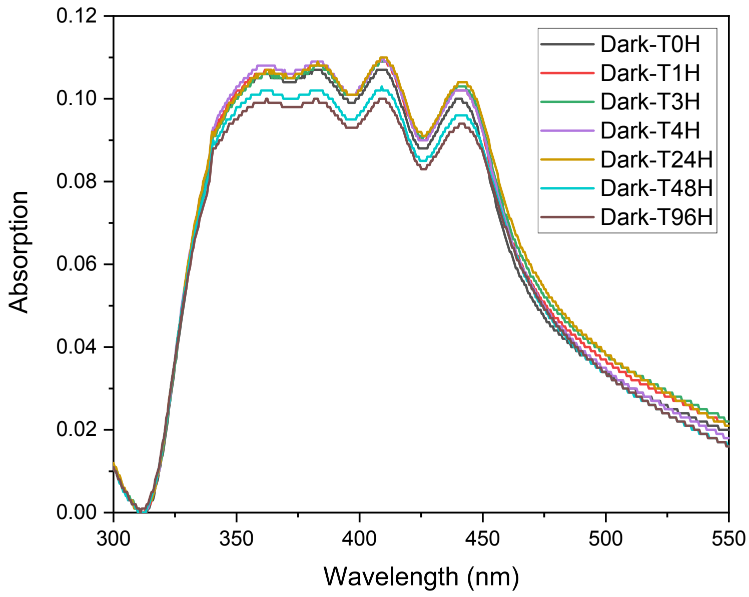

Figure S21 UV-Vis absorption spectra of DH-DTH thin films spun cast from chloroform (7mg/mL) kept in the dark and measured over time.

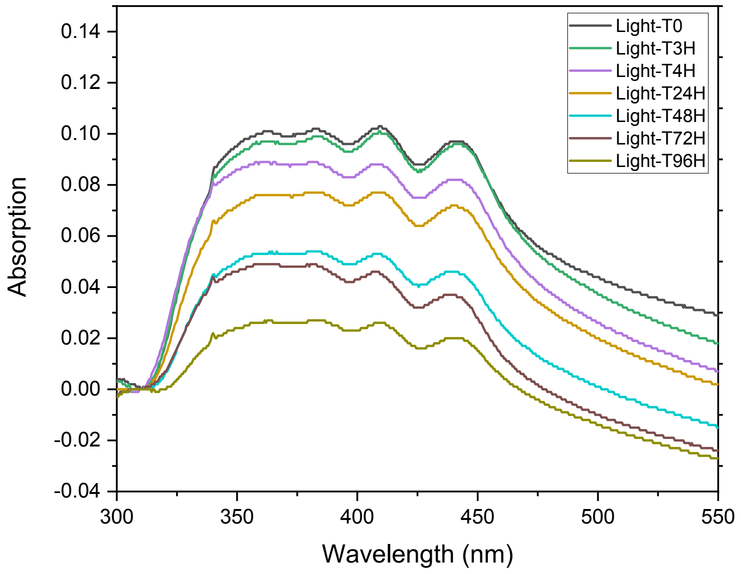

Figure S22 UV-Vis absorption spectra of DH-DTH thin films spun cast from chloroform (7mg/mL) kept in the light and measured over time.

S8 References

(1) Bensasson, R.; Land, E. J.; Lafferty, J.; Sinclair, R. S.; Truscott, T. G. The Triplet State of 1,6-Diphenyl-1,3,5-Hexatriene and 1,8-Diphenyl-1,3,5,7-Octatetraene. *Chem. Phys. Lett.* **1976**, *41* (2), 333–335. https://doi.org/https://doi.org/10.1016/0009-2614(76)80823-8.

(2) Sheldrick, G. M. {\it SHELXT} {--} Integrated Space-Group and Crystal-Structure Determination. *Acta Crystallogr. Sect. A* **2015**, *71* (1), 3–8. https://doi.org/10.1107/S2053273314026370.

(3) Sheldrick, G. M. Crystal Structure Refinement with {\it SHELXL}. *Acta Crystallogr. Sect. C* **2015**, *71* (1), 3–8. https://doi.org/10.1107/S2053229614024218.

(4) Spek, A. L. Structure Validation in Chemical Crystallography. *Acta Crystallogr. Sect. D* **2009**, *65* (2), 148–155. https://doi.org/10.1107/S090744490804362X.

(5) Clark, S. J.; Segall, M. D.; Pickard, C. J.; Hasnip, P. J.; Probert, M. I. J.; Refson, K.; Payne, M. C. First Principles Methods Using CASTEP. *Zeitschrift für Krist. - Cryst. Mater.* **2005**, *220* (5–6), 567–570. https://doi.org/doi:10.1524/zkri.220.5.567.65075.

(6) Perdew, J. P.; Burke, K.; Ernzerhof, M. Generalized Gradient Approximation Made Simple. *Phys. Rev. Lett.* **1996**, *77* (18), 3865–3868. https://doi.org/10.1103/PhysRevLett.77.3865.

(7) Grimme, S. Semiempirical GGA-Type Density Functional Constructed with a Long-Range Dispersion Correction. *J. Comput. Chem.* **2006**, *27* (15), 1787–1799. https://doi.org/https://doi.org/10.1002/jcc.20495.

(8) van de Streek, J.; Neumann, M. A. Validation of Experimental Molecular Crystal Structures with Dispersion-Corrected Density Functional Theory Calculations. *Acta Crystallogr. Sect. B* **2010**, *66* (5), 544–558. https://doi.org/10.1107/S0108768110031873.

(9) Jiang, Z. GIXSGUI: A MATLAB Toolbox for Grazing-Incidence X-Ray Scattering Data Visualization and Reduction, and Indexing of Buried Three-Dimensional Periodic Nanostructured Films. *J. Appl. Crystallogr.* **2015**, *48* (3), 917–926. https://doi.org/https://doi.org/10.1107/S1600576715004434.

(10) Lee, C.-L.; Yang, X.; Greenham, N. C. Determination of the Triplet Excited-State Absorption Cross Section in a Polyfluorene by Energy Transfer from a Phosphorescent Metal Complex. *Phys. Rev. B* **2007**, *76* (24), 245201. https://doi.org/10.1103/PhysRevB.76.245201.

(11) Fallon, K. J.; Budden, P.; Salvadori, E.; Ganose, A. M.; Savory, C. N.; Eyre, L.; Dowland, S.; Ai, Q.; Goodlett, S.; Risko, C.; Scanlon, D. O.; Kay, C. W. M.; Rao, A.; Friend, R. H.; Musser, A. J.; Bronstein, H. Exploiting Excited-State Aromaticity To Design Highly Stable Singlet Fission Materials. *J. Am. Chem. Soc.* **2019**, *141* (35), 13867–13876. https://doi.org/10.1021/jacs.9b06346.
